# A Highly Intensified ART Regimen Induces Long-Term Viral Suppression and Restriction of the Viral Reservoir in a Simian AIDS Model

**DOI:** 10.1371/journal.ppat.1002774

**Published:** 2012-06-21

**Authors:** Iart Luca Shytaj, Sandro Norelli, Barbara Chirullo, Alessandro Della Corte, Matt Collins, Jake Yalley-Ogunro, Jack Greenhouse, Nunzio Iraci, Edward P. Acosta, Maria Letizia Barreca, Mark G. Lewis, Andrea Savarino

**Affiliations:** 1 Department of Infectious, Parasitic and Immune-mediated Diseases, Istituto Superiore di Sanità, Viale Regina Elena, Rome, Italy; 2 BIOQUAL, Inc., Rockville, Maryland, United States of America; 3 Dipartimento di Chimica e Tecnologia del Farmaco, Facoltà di Farmacia, Università di Perugia, Perugia, Italy; 4 The University of Alabama at Birmingham, Division of Clinical Pharmacology, Birmingham, Alabama, United States of America; Emory University, United States of America

## Abstract

Stably suppressed viremia during ART is essential for establishing reliable simian models for HIV/AIDS. We tested the efficacy of a multidrug ART (highly intensified ART) in a wide range of viremic conditions (10^3^–10^7^ viral RNA copies/mL) in SIVmac251-infected rhesus macaques, and its impact on the viral reservoir. Eleven macaques in the pre-AIDS stage of the disease were treated with a multidrug combination (highly intensified ART) consisting of two nucleosidic/nucleotidic reverse transcriptase inhibitors (emtricitabine and tenofovir), an integrase inhibitor (raltegravir), a protease inhibitor (ritonavir-boosted darunavir) and the CCR5 blocker maraviroc. All animals stably displayed viral loads below the limit of detection of the assay (*i.e*. <40 RNA copies/mL) after starting highly intensified ART. By increasing the sensitivity of the assay to 3 RNA copies/mL, viral load was still below the limit of detection in all subjects tested. Importantly, viral DNA resulted below the assay detection limit (<2 copies of DNA/5*10^5^ cells) in PBMCs and rectal biopsies of all animals at the end of the follow-up, and in lymph node biopsies from the majority of the study subjects. Moreover, highly intensified ART decreased central/transitional memory, effector memory and activated (HLA-DR^+^) effector memory CD4^+^ T-cells *in vivo*, in line with the role of these subsets as the main cell subpopulations harbouring the virus. Finally, treatment with highly intensified ART at viral load rebound following suspension of a previous anti-reservoir therapy eventually improved the spontaneous containment of viral load following suspension of the second therapeutic cycle, thus leading to a persistent suppression of viremia in the absence of ART. In conclusion, we show, for the first time, complete suppression of viral load by highly intensified ART and a likely associated restriction of the viral reservoir in the macaque AIDS model, making it a useful platform for testing potential cures for AIDS.

## Author Summary

Novel research aimed at finding a cure for AIDS requires animal models responding to human antiretroviral drugs. However, there have been few antiretrovirals cross-active against the simian viruses. In this study, we expanded the arsenal of drugs active against the simian retrovirus SIVmac251 and showed that this virus is inhibited by the protease inhibitor, darunavir, and the CCR5 blocker, maraviroc. Administration of these two drugs in combination with the reverse transcriptase inhibitors, tenofovir and emtricitabine, and the integrase inhibitor, raltegravir, resulted in prolonged plasma viral loads below assay detection limits, and, surprisingly, restricted the viral reservoir, a marker of which is viral DNA. We then decided to employ this multidrug regimen (termed “highly intensified ART”) in order to increase the potency of a previous strategy based on the gold drug auranofin, which recently proved able to restrict the viral reservoir *in vivo*. A short course of highly intensified ART following the previous treatment resulted, upon therapy suspension, in a remarkably spontaneous control of the infection, that may pave the way to a persistent suppression of viremia in the absence of ART. These results corroborate the robustness of the macaque AIDS model as a vanguard for potentially future treatments for HIV in humans.

## Introduction

The study of persistence of viral sanctuaries during antiretroviral therapy (ART) and the possibility for their therapeutic targeting is crucial for eradication of HIV-1. Animal models for lentiviral persistence during therapy are therefore needed. The creation of such animal models requires knowledge of the response of animal lentiviruses to antiretroviral drugs adopted in treatment of humans with HIV-1. Finding cross-active drugs has been a difficult task because non-HIV-1 lentiviruses often mimic drug resistance mutations found in HIV-1. This mimicry has been shown for the viral protease [1] and for the portion of reverse transcriptase (RT) that binds the non-nucleosidic reverse transcriptase inhibitors (NNRTIs) [2].

One of the current models is based on macaques infected with a molecularly engineered simian immunodeficiency virus (SIVmac239) expressing HIV-1 RT, in order to overcome drug resistance mimicry of the primate lentiviruses to NNRTIs [3]. Another model (SIV-based) has been developed for neurotropic infection, a condition often occurring in late-stage AIDS [4]. In this case, in order to by-pass the different response to antiretrovirals, the authors used a drug combination which is not adopted in humans. However, in both of these animal models, low-level viremia persisted and viral RNA was consistently detectable in anatomical sanctuaries [3], [4].

A model recently developed by our group is based on a polyclonal virus, such as SIVmac251, mimicking, at least in part, the genetic diversity of HIV-1 naturally inoculated in human subjects [5]. It was recently shown that SIVmac251 responds to combined ART consisting of two nucleosidic/nucleotidic reverse transcriptase inhibitors (NRTIs), *i.e.* tenofovir and emtricitabine, and the integrase inhibitor raltegravir [5], [6]. In this treatment model, the virus persists during ART, and viral load rebounds following treatment suspension in a time frame remarkably similar to that observed in humans after treatment interruption [7].

Recent research has added more credit to the macaque AIDS model, showing that, similarly to humans [8], [9], rhesus macaques (*Macaca mulatta*) harbour a central memory CD4^+^ T-cell reservoir, which plays a pivotal role in AIDS pathogenesis [7], [10]. Important insight has been derived from comparisons between rhesus macaques and sooty mangabays (*Cercocebus atys*) which, unlike *M. mulatta*, do not progress to AIDS [11]. *M. mulatta*, but not *C. atys*, shows up-regulation of the lentiviral co-receptor CCR5 in activated central memory T-cells, thus rendering this T-cell pool highly permissive to infection [10]. Conversely, the reduction of the long-lived memory T-cells (CD95^+^CD28^+^), including central memory T-cells, by the gold-based compound auranofin in intensified ART (iART)-treated rhesus macaques resulted in decreased levels of viral DNA and delayed progression of the infection upon therapy suspension [7]. Therefore, a model mimicking the effects of suppressive ART in humans is of fundamental importance also for the study of the dynamics of this viral reservoir.

One major limitation of current models for HIV persistence during therapy is their large discrepancy from conditions observed in humans. So far, due to financial and temporal constraints, animals have been chosen from homogeneous cohorts in terms of timing, type and route of the inocula, and have been treated in the early phases of chronic infection [3]–[6] or during acute infection [12]. Instead, at therapy initiation, HIV-infected humans are usually characterized by different timings and routes of disease acquisition and different levels of progression of the infection. In order to obtain a robust animal model for HIV persistence during therapy, the drug regimens should display similar efficacies as compared to those employed for human treatment, and reproducible control of heterogeneous viral loads in wide cohorts of subjects with different characteristics and previous treatment histories.

Here, we report a highly intensified ART (H-iART) regimen for the simian model, reproducibly capable of decreasing viral load to levels below assay detection limits in SIVmac251-infected macaques starting from a wide range of baseline viremic conditions, and overcoming previous treatment failures. We also report an unexpectedly impressive restriction of viral DNA in peripheral blood mononuclear cells, obtained by means of a pharmacological strategy entirely based on antiretroviral drugs.

## Materials and Methods

### Cells and virological assays

CEMx174 and HTLV-I-transformed MT-4 cells were grown in RPMI-1640 medium supplemented with glutamine (200 µg/mL) (Invitrogen Life Technologies, Inc. Carlsbad, California), 10% heat-inactivated fetal bovine serum (FBS; Invitrogen Life Technologies), penicillin (500 U/mL; Pharmacia Italia SPA) and streptomycin (66.6 U/mL; Bristol-Myers, Sermoneta, LT).

Peripheral blood from uninfected nonhuman primates was diluted 1∶2 with PBS 1x-NaCl, and peripheral blood mononuclear cells (PBMCs) were Ficoll-separated, resuspended at a concentration of 2×10^6^/mL and stimulated for 3 days with 5 µg/mL phytohaemoagglutinin (PHA) (Difco Laboratories, Detroit, MI, USA) and 100 units/mL of human recombinant IL-2 (Roche Diagnostics, Indianapolis, IN, USA).

CEMx174, MT-4 cells, and three-day old PBMCs were challenged with standard viral stock preparations for 2 hours in an incubator at 37°C with 5% CO_2_, washed and incubated with increasing drug concentrations (0.0001–1 µM), according to a previously published protocol [5]. The assays on virus entry inhibitors such as maraviroc (MRV), were conducted as in [13]. Briefly, the drug was first added during incubation with the virus and the same drug concentrations were then re-added upon cell washing. In MT-4 cells, through the MTT assay (MT4-MTT), we measured inhibition of the cytopathic effect of the two viruses. The assay was performed when the majority of control infected cells were dead. At different intervals post-infection, the viral core antigen p27 was measured in supernatants by antigen-capture ELISA assays (Advanced BioScience lab., Inc.).

### Animal treatment

The Indian rhesus macaques used in this study were housed at Bioqual, Inc., according to standards and guidelines as set forth in the Animal Welfare Act, the Guide for the Care and Use of Laboratory Animals, and the Association for the Assessment and Accreditation of Laboratory Animal Care (AAALAC), following approval by the Institutional Animal Care and Use Committee (IACUC). A total of eleven macaques have been enrolled for this study, while five previously enrolled macaques have been employed as historical controls.

For the pilot study, four SIVmac251-infected non-human primates (*M. mulatta*) that had been stably viremic at least for the last 3.3 months were put under a regimen (*i.e.* ART) consisting of tenofovir (PMPA), emtricitabine (FTC) and raltegravir [5], for 1.5 months. To improve control of viral load, this regimen was intensified by the addition of darunavir (DRV) boosted with ritonavir (/r) [intensified ART (iART)]. After 80 days, the treatment was further reinforced [highly intensified ART (H-iART)] by the addition of maraviroc.

For the second part of the study, eight additional SIVmac251 infected animals were used. These animals were divided into three treatment groups. One group (n = 2) was treated with MRV/r alone for three weeks, followed by addition of tenofovir/emtricitabine/raltegravir/DRV. A second group (n = 4) was treated with all H-iART drugs administered simultaneously. A third group (n = 2) was treated with iART to serve as controls.

For the combined antireservoir/antiretroviral therapeutic protocols, macaque P252, previously treated with iART plus the anti-reservoir drug auranofin (for detail, see Ref. 7), was put under a H-iART regimen for one month when viral load rebounded after suspension of the previous treatment. Another macaque, P177 of the pilot study, was treated (after the end of the follow-up aimed at monitoring the effects of H-iART alone) with auranofin in addition to H-iART. This macaque was then subjected, similarly to P252, to a further cycle of H-iART at viral load rebound.

More detailed information on the macaques enrolled, their viro-immunological background and the therapeutic regimens adopted for each animal can be found in Table S1.

All animals were dosed subcutaneously with tenofovir, and emtricitabine, and orally (with food) with raltegravir, DRV/r, and MRV. Initial drug dosages were: tenofovir, 30 mg/kg/day; emtricitabine, 50 mg/kg/day; raltegravir, 100 mg bid; DRV, 375 mg bid (for macaques starting from viral loads lower than 10^5^ viral RNA copies/mL) or 700 mg bid (for macaques starting from viral loads higher than 10^5^ viral RNA copies/mL); ritonavir 50 mg bid; MRV 100 mg bid.

Tenofovir and emtricitabine were kindly provided by Gilead Sciences (Foster City, CA). Raltegravir, DRV/r and MRV were purchased from the manufacturers.

### Quantitative assay for SIVmac251 viral RNA levels

For measurement of plasma SIVmac251 RNA levels, a quantitative TaqMan RNA reverse transcription-PCR (RT-PCR) assay (Applied Biosystems, Foster City, Calif.) was used, which targets a conserved region of the *gag* transcripts. The samples were then amplified according to a method previously validated in our hands [see ref 5 and Fig. S1]. The sensitivity of the method is two copies per run, which results in a detection limit as low as 40 RNA copies/mL in our routine analyses. Briefly, a 500-µL aliquot of plasma was spun down at 13,000× *g* for 1 h. The liquid was poured off and 1 mL of RNA-STAT 60 was added. After 5 min., 250 µL of chloroform was added and vortexed. The samples were spun at the same speed for 1 h. The clear aqueous layer on top was removed, and added to 500 µL of isopropanol. Then, 10 µl of 10 µg/mL tRNA was added and precipitated overnight at −20°C. The samples were spun for 1 hour, washed with a cold (−20°C) 75% ethanol solution, and re-spun for 1 h. The RNA was resuspended in 30 µL of RNAse-free water. 10% of the resuspended RNA was added to Taqman reagents (Applied Biosystems), plus primers and probe, and amplified in a 7700 Sequence Detection System by Applied Biosystems. Briefly, the sample was reverse transcribed at 48°C for 30 min. using One-Step RT-PCR Master Mix (Applied Biosystems), then held at 95°C for 10 min., and run for 40 cycles at 95°C for 15 sec. and 60°C for 1 min. The following PCR primer/probes were used: SIV2-U 5′ AGTATGGGCAGCAAATGAAT 3′ (forward primer), SIV2-D 5′ GGCACTATTGGAGCTAAGAC 3′ (reverse primer), SIV-P 6FAM-AGATTTGGATTAGCAGAAAGCCTGTTGGA-TAMRA (TaqMan probe). The signal was finally compared to a standard curve of known concentrations from 10^7^ down to 1 copy (the linear range of concentration/signal relation spans eight *Log*s). All samples were done in triplicate for consistency and accuracy. In our increased sensitivity analyses, RNA was extracted from 6 mL of starting plasma, leading to a sensitivity threshold of 3 copies/mL. The inter-assay variability of the assay is 23.4%; The intra-assay variability is 20.6%.

### Quantitative assay for SIVmac251 proviral DNA

For proviral DNA detection, cells were spun down to a pellet, and the supernatant was poured off. The cell pellet was lysed with 1 mL of DNASTAT for 10 min. 250 µL of chloroform was added and the mixture was vortexed. The samples were spun at 13,000 for 1 h. and the aqueous layer was removed and added to another tube. To this, 500 µL of isopropanol was added, and the mixture was precipitated overnight at −20°C. The samples were then spun for 1 h and the precipitate was washed with a −20°C-cold, 75% ethanol solution, and re-spun for 1 h. The DNA pellet was resuspended in 30 µL of water and 10% of the resulting solution was added to Taqman reagents (Applied Biosystems) plus primers and probe (the same as in previous paragraph) and amplified in a 7700 Sequence Detection System by Applied Biosystems. The signal was finally compared to a standard curve of known concentrations from 10^6^ down to 1 copy (the linear range of concentration/signal relation spans seven *Log*s). The detection limit of this assay is two copies of proviral DNA/5×10^5^ cells. The inter-assay variability is 28.3%; the intra-assay variability is 9.9%.

The presence of PCR inhibitors in both the quantitative assays (viral RNA and proviral DNA) has been ruled out by spiking the samples with known amounts of viral RNA and proviral DNA respectively (see Table 1 and Table S2).

**Table 1:** Ultrasensitive viral load measurements.

Macaque	Time (in days)	Viral RNA (copies/mL)	Spike copies original count (RNA copies/mL)
**P157**	204 and 239 (see Fig. 2)	<3	* 7008
**P185**	204 and 239 (see Fig. 2)	<3	* 10822
**P188**	204 and 239 (see Fig. 2)	<3	* 8194
**P177**	312 and 347 (see Fig. 8)	<3	* 7692
	**§**591 and 599 (see Fig. 8)	<3	** 6062
	**§**654 and 658 (see Fig. 8)	<3	** 9060
**P252**	**§**215 and 365 (see Fig. 8)	<3	** 6201
**4416**	238 and 272 (see Fig. 4)	<3	* 8276
**BD12**	98 and 105 (see Fig. 4)	<3	** 9319
**BD53**	98 and 105 (see Fig. 4)	<3	** 8494
**BD64**	400 and 407 (see Fig. 4)	<3	* 18997
**BD69**	381 and 388 (see Fig. 4)	27	* 8900
	429 and 437 (see Fig. 4)	<4 (less plasma)	** 5569
	451 and 458 (see Fig. 4)	<3	*** 20

Shown are the real time PCR viral RNA measurements of pooled plasma samples (two pooled samples per measurement; total plasma volume 6 mL; detection limit = 3 RNA copies/mL). The time points selected for the analyses are shown as days from the zero point adopted in figures 2, 4 and 8. In these time points, the macaques were under H-iART (unmarked), or off-treatment after therapy suspension (marked with “§”). As a control of the assay variability and to exclude PCR inhibition, spiked RNA measurements for each plasma sample are shown. The RNA copy numbers (in copies/mL) used for the spiked measurements were: * 8686; ** 8605; *** 34. The low copy number spike was chosen to double-check the absence of low-level viremia in macaque BD69 that was the only one showing detectable viral RNA during the first PCR run.

### Drug concentrations in plasma (DRV and MRV)

Animals were bled before feeding in the morning, in order to obtain reliable measurements of trough drug levels. Plasma was obtained from supernatants of ficoll-centrifuged blood.

For DRV, sample preparation involved addition of an internal standard and liquid-liquid extraction with 2 mL *tert*-butylmethylether (*t*BME) at basic pH, and reconstitution in 100 µL of mobile phase to concentrate the sample. Reversed phase chromatographic separation of the drugs and internal standard was performed on a YMC, C8 analytical column under isocratic conditions. A binary mobile phase was used consisting of 55% 20 mM sodium acetate buffer (pH 4.88) and 45% acetonitrile. The UV detector set to monitor the 212 nm wavelength provided adequate sensitivity with minimal interference from endogenous matrix components. Calibration curves are linear over the range of 50 to 20,000 ng/mL. Inter- and intraday variability was less than 10%.

For MRV, a protein precipitation method using acetonitrile (AcN) containing internal standard (MVC-d6) was employed to extract the drug from macaques' plasma. An aliquot of the supernatant was further diluted with 0.5% tirfluoroacetic acid to maintain signal intensity within the linear range of the instrument. Reversed phase chromatographic separation was performed on an XBridge C18 analytical column under isocratic conditions. A binary mobile phase consisting of 0.1% formic acid in water and 0.1% formic acid in acetonitrile (72∶28) was used and provided adequate separation from other analytes. Detection and quantitation was achieved by multiple reaction monitoring (MRM), and MVC and internal standard were detected using the following transitions for protonated molecular products [M+H]^+^: *m/z* MVC 514.2>106.0; *m/z* MVC-d6 520.3>115.0. The assay has a dynamic range of 5 to 5,000 ng/mL using 20 µL plasma.

For both DRV and MRV total drug concentrations were measured (*i.e* free and protein bound).

### Immunofluorescent staining and flow-cytometric analysis

Hematological analyses were performed by IDEXX (IDEXX Preclinical Research, North Grafton, MA). For calculation of absolute CD4^+^ and CD8^+^ T-cell numbers, whole blood was stained with anti-CD3-fluorescein isothiocyanate (FITC)/anti-CD4-phycoerythrin (PE)/anti-CD8-peridinin chlorophyll α protein (PerCP)/anti-CD28-allophycocyanin (APC), and anti-CD2-FITC/anti-CD20-PE, and red blood cells were lysed using lysing reagent (Beckman Coulter, Inc., Fullerton, Calif.). Samples were run on a FACSCanto II (BD Biosciences, San Jose, CA).

Staining for naïve (T_N_: CD28^+^CD95^−^), central and transitional memory (T_TCM/TM_: CD28^+^CD95^+^), and effector memory (T_EM_: CD28^−^CD95^+^) T-cells was performed on PBMCs isolated from total blood of three rhesus macaques treated with H-iART. For each animal, the blood was collected monthly from 0 to 4 months from the addition of MRV to the drug regimen. The cells (3×10^5^ per sample) were surface stained by incubation with six appropriately titrated monoclonal antibodies (mAbs) for 20′ at 4°C, washed with PBS and resuspended in 1% paraformaldehyde in PBS. The following mAbs were used: anti-CD3 (APC-Cy7), anti-CD4 (Per-CP), anti-CD8 (Pe-Cy7), anti-CD20 (APC), anti-CD28 (FITC) and anti-CD95 (PE). Six-parameter flow-cytometric analysis was performed on a FACS Canto II instrument (BD Biosciences) [7]. Staining for HLA DR^+^ T-cells was performed with the same procedure described above, but with the substitution of an anti-HLA-DR antibody (APC, clone G46-6) to the aforementioned anti-CD20 antibody. The absolute numbers of naïve (CD95^−^CD28^+^), long-lived (CD95^+^CD28^+^) and short-lived (CD95^+^CD28^−^) memory CD4^+^ T-cells and the numbers of HLA-DR^+^ cells were deduced from percentage values of parent cells.

### SIVmac251 specific cellular immunity-ELISPOT assay

Specific immune responses were detected by measuring gamma interferon (IFN-γ) secretion of macaque PBMCs stimulated with a SIVmac239 Gag peptide (15-mer, obtained through the AIDS Research and Reference Reagent Program, National Institutes of Health [NIH], catalogue no. 6204, peptide 64) in an enzyme-linked immunospot (ELISPOT) assay. The assay was performed with the ELISpotPRO for monkey interferon-γ kit (Mabtech AB, Nacka Strand, Sweden) according to the manufacturer's instructions. Briefly, 1.5×10^5^ Ficoll isolated macaque PBMCs were added to 96 well plates pre-coated with an anti-human/monkey IFN-γ antibody (MAb GZ-4). Cells were resuspended in RPMI 1640+10% FBS with 2 µg/mL of the peptide. After 48 hours incubation at 37°C with 5% CO^2^, the cells were rinsed from the plates, and a biotinylated anti-human/monkey IFN-γ antibody (MAb 7-B6-1; Mabtech) was added to the wells. The plates were then washed with PBS and incubated with the substrate solution (BCIP/NBT-plus). Spots were counted by using an automated reader (Immunospot Reader, CTL analyzers, LLC, Cleveland, OH). Numbers of spot-forming cells (SFC)/10^6^ cells for each set of wells were averaged. A response was considered positive if the number of SFC/10^6^ cells was at least four times the background value.

### Statistical and biomathematical analyses

Data were analyzed using the software GraphPad Prism 5.00.288 (GraphPad Software, Inc., San Diego, CA). For calculation of the EC_50_ and EC_90_ values, data were transformed into percentage-of-inhibition values, plotted on *x,y* graphs, and subjected to linear or non-linear regression, depending on the best-fitting equation. Response to drugs *in vivo* was evaluated by repeated-measures ANOVA, followed by an appropriate post-test to analyze differences between time points. An appropriate transformation was employed to restore normality, where necessary. *Logit* analysis was adopted to investigate the influence of variables on binary outcomes, using an online calculator (http://statpages.org/logistic.html).

Trends in time were analyzed by regression analysis (GraphPad Prism), using the most appropriate equations. Akaike's information criteria (AICc) were used to select the model that was most likely to have generated the data and to compare the differences between equation parameters.

The inter-assay variability of quantitative real time PCR was estimated as an average of the coefficients of variation (CV) of matched measurements in two assays conducted on different occasions; the intra-assay variability was estimated as the coefficient of variation of multiple replicates (at least five) within the same assay.

Numerical simulations were performed with the ordinary differential equations solver ODEPACK of the Scilab 5.3.3 software (http://www.scilab.org/). The solver is based on finite difference methods for non-stiff problems, but it dynamically monitors the data in order to decide whether the stiffness of the problem requires a Backward Differentiation Formula method. The values of the discrete five-dimensional vector function of the solution were computed every 0.01 days. Details on mathematical modeling are given in Text S1.

## Results

### SIVmac251 is susceptible to darunavir (DRV) and maraviroc (MRV)

**Figure 1:**
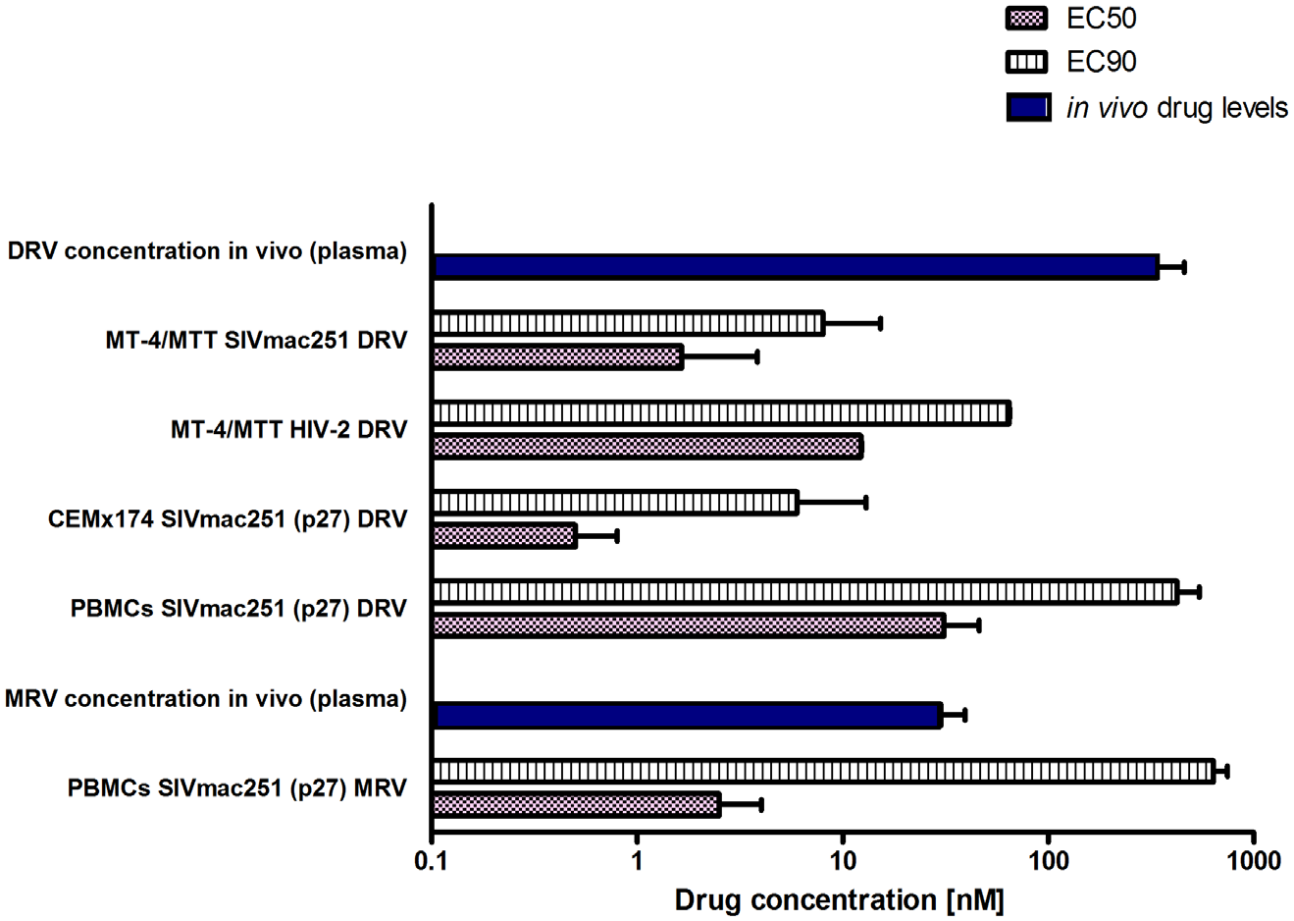
SIVmac251 is susceptible to DRV and MRV. Comparison between effective drug concentrations required for 50% and 90% inhibition of viral replication *in vitro* (respectively, EC_50_ and EC_90_) and the *in vivo* total levels (*i.e.* free and protein bound) of DRV and MRV in the plasma of six animals treated with H-iART. All values are displayed as mean + SEM.

The first part of this study was aimed at obtaining long-term viral suppression in a group of macaques (n = 4) in order to develop a suitable platform for testing experimental eradication strategies. We first analyzed the susceptibility of SIVmac251 to the protease inhibitor darunavir (DRV) and the CCR5 blocker maraviroc (MRV) in order to expand the arsenal of antiretroviral options available for the macaque AIDS model. DRV was chosen because of its well documented ability to inhibit several drug-resistant HIV-1 isolates as well as HIV-2, a virus closely related to SIVmac251 [1], [14], [15]. Moreover, the choice of this drug was supported by preliminary bioinformatic and molecular modeling analyses showing the potential interactions of DRV with the SIVmac251 protease [Text S2 and Fig. S2]. MRV, a CCR5 antagonist, was chosen on the basis of the important role of CCR5 as a SIVmac251 co-receptor [16] and due to the antilentiviral activity previously demonstrated by one experimental CCR5 blocker in macaques [17]. Moreover, retrospective analysis of one previous *in-vivo* experiment supported the anti-SIVmac251 effect of this drug [Text S3 and Fig. S3]. Results from tissue culture experiments showed that both DRV and MRV inhibited SIVmac251 replication in the nanomolar range, with EC_50_ values well below the trough concentrations detected in macaques treated with these drugs and described below in the text. (Fig. 1).

### DRV improves the virological response of SIVmac251-infected macaques to ART

**Figure 2:**
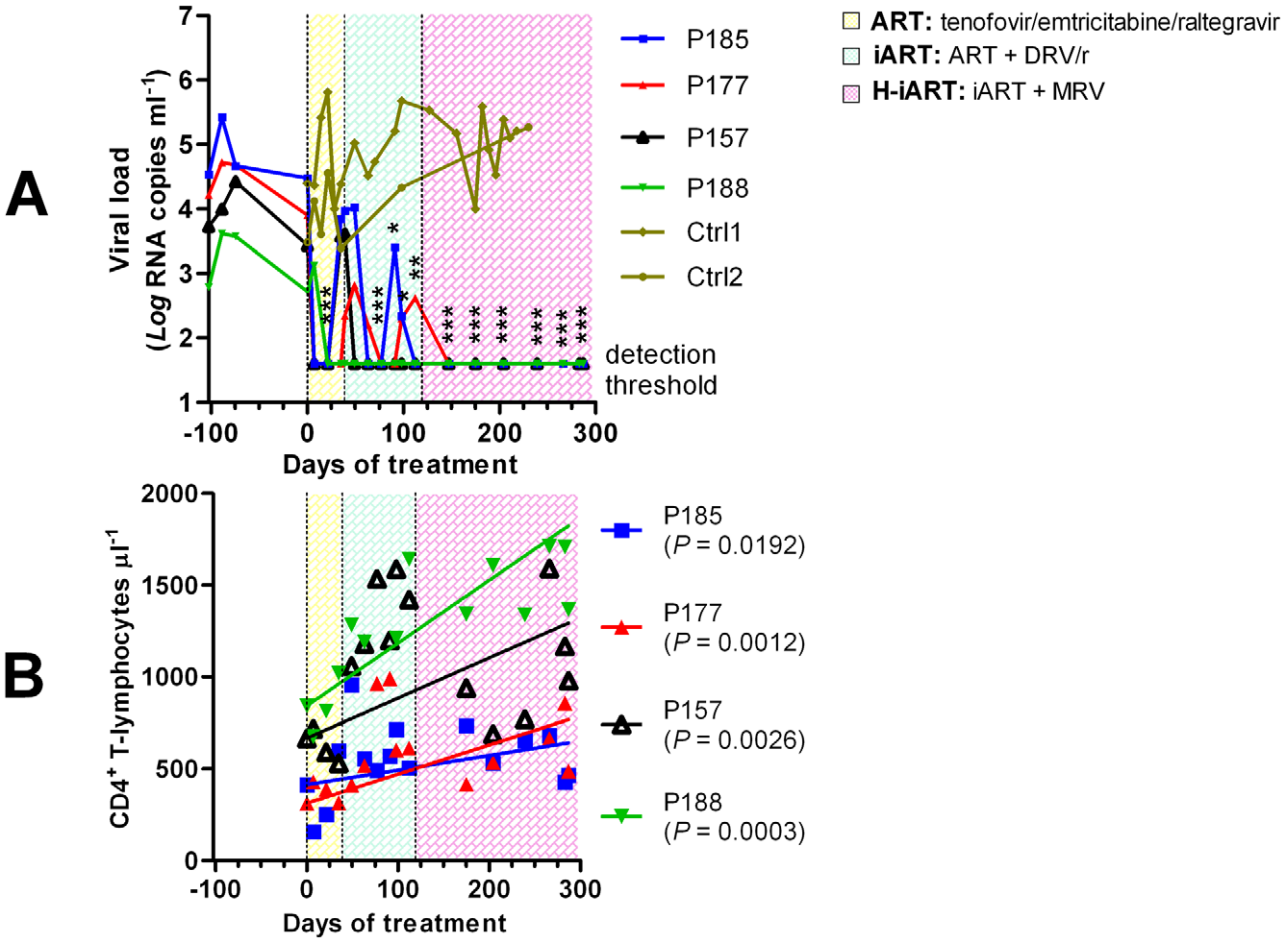
Viro-immunological control of antiretrovirally treated macaques chronically infected with SIVmac251. Panel A: Plasma viral loads. Panel B: CD4 counts. The sequential treatments are represented by the colored areas. Asterisks represent the significant differences from baseline values (respectively _*_ *P*<0.05; _**_ *P*<0.01; _***_ *P*<0.001), as calculated by Bonferroni's test. The values corresponding to the different macaques, whose denominations are given in the legends, are shown by the different symbols and connecting lines. As a comparison, panel A shows the viral loads dynamics of two untreated macaques (olive).

A group of macaques [n = 4] displaying signs of immune deterioration (eighteen months post-inoculation) was treated with a regimen of tenofovir, emtricitabine and raltegravir (Fig. 2). These macaques were derived from viral titration experiments and selected among those maintaining stable plasma viral loads (Fig. 2A). The selected animals displayed viral load set points between 10^3^ and 10^5^ viral RNA copies/mL. As our study was aimed at obtaining a model mimicking the conditions found in HIV-1-infected individuals under ART, such baseline values were chosen in order to reflect the average viral loads at which treatment is started in humans. The CD4 counts displayed by the macaques enrolled in this “pilot” study were significantly lower than values observed in uninfected controls (Fig. S4), suggesting that they were unlikely to be long-term non-progressors or *élite* controllers.

The three-drug regimen proved insufficient to maintain control of viral load in three of the four animals treated (Fig. 2A). DRV (375 mg bid), boosted with ritonavir (50 mg bid), henceforth referred to as DRV/r, was added to the treatment in an attempt to obtain a more stable control of viral load. DRV/r significantly improved control of viral load, inasmuch as viral RNA in plasma was maintained at a significantly lower level as compared to the pre-therapy values (Fig. 2A). No similarly decreasing trend of viral load was observed in an untreated control group of macaques [n = 2] showing non-significant differences in baseline viral loads as compared to the treatment group (two tailed *t*-test: *P* = 0.803; Fig. 2A). We conclude that the iART regimen adopted improves control of viral load in SIVmac251-infected macaques.

### A H-iART regimen induces a prolonged control of residual viremia

To increase the chances for long-term control of SIVmac251 replication, we explored the *in-vivo* efficacy of the CCR5 inhibitor MRV. This drug (100 mg BID) was eventually added to the drug cocktail in the aforementioned group of macaques (Fig. 2). After MRV was started, all macaques stably maintained viral loads below the limit of detection of the assay (*i.e.* 40 copies RNA/mL; Fig. 2A). There were also significant increases in the absolute numbers of CD4^+^ T-lymphocytes (Fig. 2B). Henceforth, this multidrug combination will be referred to as highly intensified ART (H-iART).

### MRV exerts antiretroviral effects in vivo

**Figure 3:**
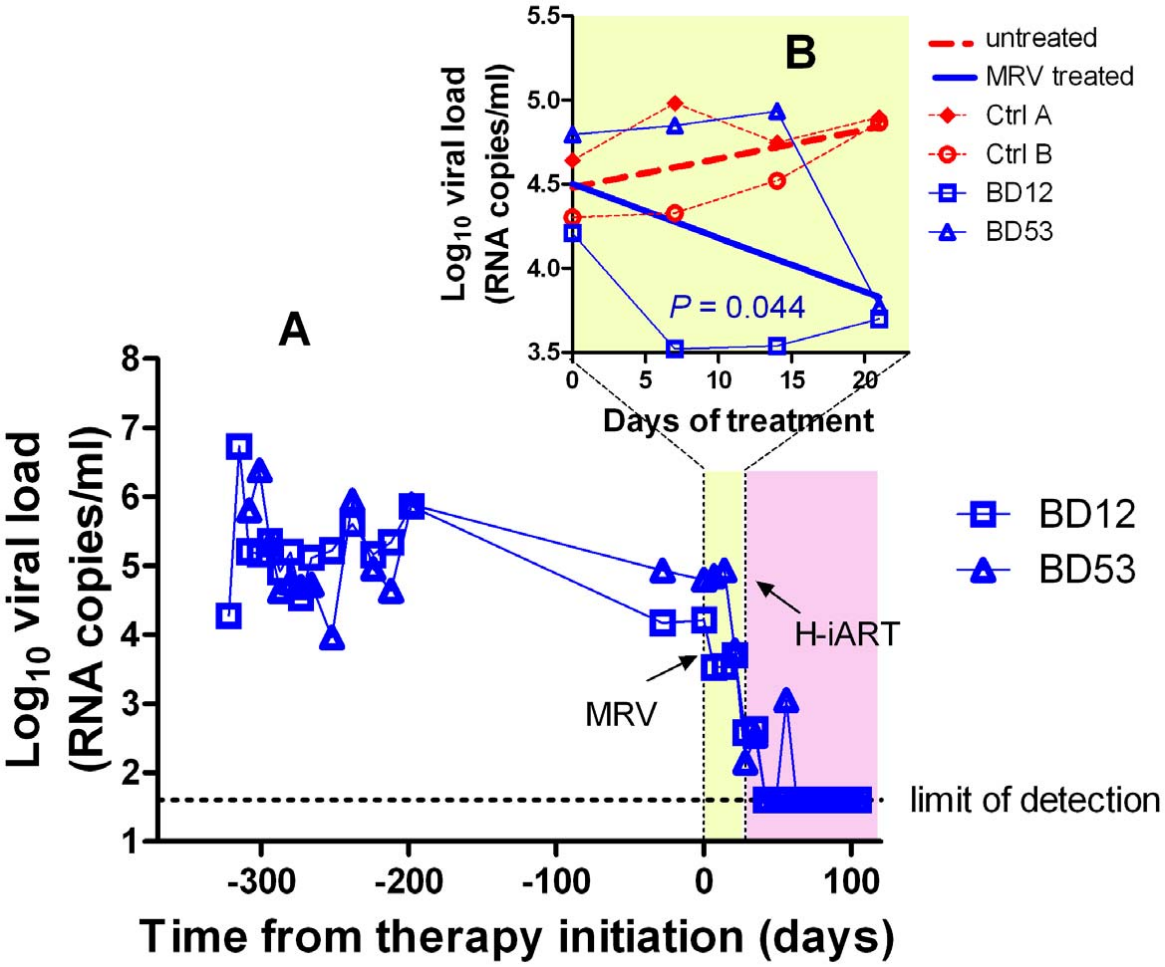
Ritonavir-boosted MRV (MRV/r) is able to decrease viremia *in vivo*. Panel A: Viral load measurements after infection and during treatment with MRV/r (light yellow) and H-iART (light purple). Panel B (detail): Viral loads during MRV/r monotherapy in comparison to viral loads of untreated controls.

In order to further support the contribution of MRV to the antiretroviral effects observed, we treated two macaques with MRV (ritonavir boosted, MRV/r) in monotherapy (Fig. 3). In line with its CCR5-blocking ability, MRV decreased the viral loads in two drug-naïve macaques with dynamics similar to those previously shown by an investigational CCR5 blocker [17]. When the other H-iART drugs were added to MRV, a quick abatement of viral load to levels below the assay detection limit could be demonstrated (Fig. 3).

### H-iART suppresses viremia in a broad range of viremic conditions

**Figure 4:**
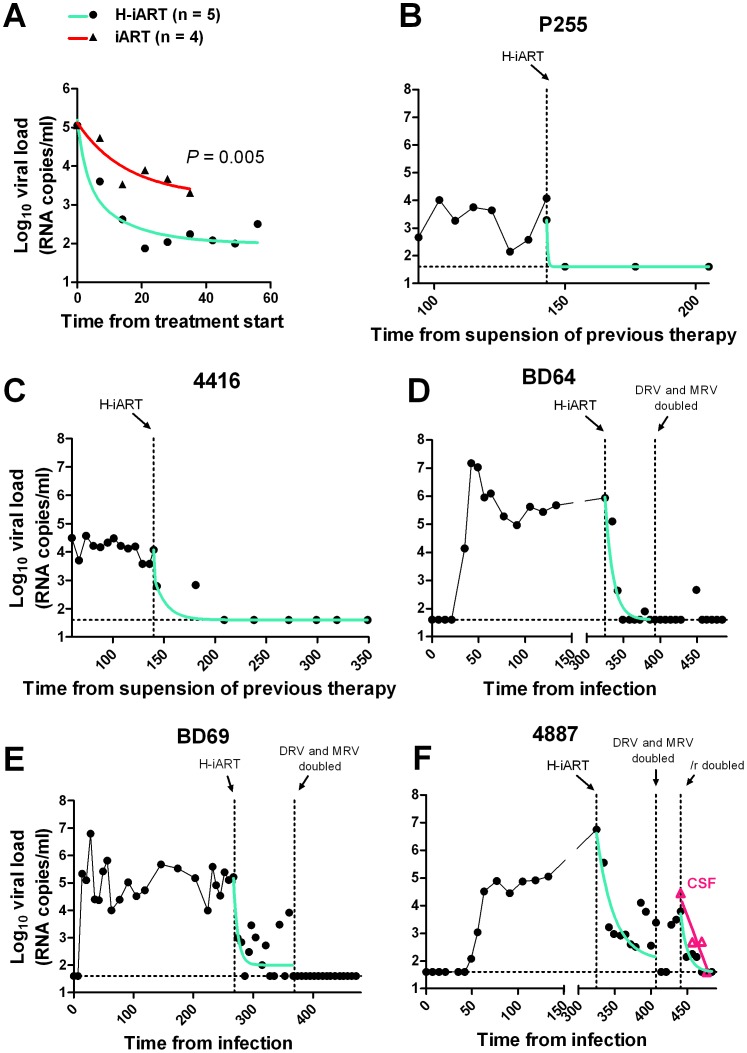
Viral load decay dynamics under H-iART treatment. Panel A: Comparison (two tailed *t*-test) of the antiretroviral efficacy of H-iART and iART. Viral loads are the mean values from 5 animals (H-iART) or 4 animals (iART). Two of the animals treated with iART are historical controls (for more detail see Table S1). The *P*-value was calculated by Aikaike's information criteria (AIC) for comparison of curves. Panels B, C, D, E, F: Nonlinear regression analysis (two phase decay) of viral load measurements during time. For macaque 4887, viral RNA levels in cerebrospinal fluid (CSF) are also shown (in magenta).

Prior to treatment with antiretrovirals, approximately one third of the experimental infections of macaques with SIVmac251 results in viral set points comparable to those displayed by the macaques described in the previous paragraphs (Fig. S5). To check whether H-iART might reproducibly control viral replication in SIVmac251 infected macaques characterized by higher viral loads, five animals with viral set points ranging from 10^3^ to 10^7^ viral RNA copies/mL of plasma were treated with H-iART, and the viral decay dynamics were compared with those of macaques treated with iART. Results clearly showed that H-iART induced a significantly more rapid decay in viral load than did iART (Fig. 4A). In line with the efficacy of H-iART, CD4^+^ T-cells increased in all study macaques (Fig. S6). We conclude that MRV-containing H-iART is superior to iART in abating viremia load in a group of macaques with a wide array of baseline viral loads.

### The extent of suppression of viral replication is dependent on baseline viral loads and drug dosage in H-iART-treated macaques

We then analyzed the viral load decay dynamics in macaques treated with H-iART *ab-initio*. SIVmac251-infected macaques responded to administration of H-iART with a two phase exponential decay, as described in humans treated with suppressive ART [18] (Fig. 4). Similarly to the average treatment outcomes in humans [19], the level of viral load suppression depended on the baseline viral loads, with macaques starting from higher viral loads showing viral blips or residual, though markedly decreased (>3 *Logs*), viral replication (Fig. 4D–F).

We increased the DRV and MRV dosage in macaques 4887, BD64 and BD69, *i.e.* those starting from higher baseline viral loads (>10^5^) and showing incomplete control of viral replication or major blips. Results showed that the improved drug regimen led to viral loads consistently below the assay detection limit in animals BD64 and BD69 (Fig. 4D,E). The increased drug dosage was also able to decrease the amplitude of the remaining sporadic blips (Fig. 4E). The resulting blips were lower than 10^3^ copies of viral RNA/mL, thus mimicking those observed in humans under ART [20]. Nevertheless, one animal (4887) experienced a further viremic episode. Analysis of the cerebrospinal fluid (CSF) of this animal showed a viral load approximately one order of magnitude higher than that in plasma, while CSF samples were below the assay detection limit (*i.e.* 40 copies/mL) in the macaques showing stable control of viral replication (data not shown). This evidence suggested that the central nervous system (CNS) was a likely major source for the rebounding virus in macaque 4887. According to previously published studies: 1) virus levels in the CSF during the advanced stages of the disease are mostly due to CNS sources [21], and 2) the protease inhibitors (*i.e.*, the only drug class in our cocktail acting at a post-translational level, and hence on chronically infected cells) are extruded from the CNS by P-glycoprotein (P-gp) molecules in the blood-brain barrier [22]. We thus intensified the P-gp blockade by increasing, from 50 to 100 mg bid, the dosage of ritonavir, which is a well-known P-gp inhibitor [23]. The viral load decreased in both plasma and CSF, with a more rapid decay kinetic in plasma, in which viral RNA eventually fell to levels below the assay detection limit (Fig. 4F). This result is in good agreement with the hypothesis of the CNS as a major source for the rebounding virus.

We conclude that macaques starting from high viral loads respond to H-iART similarly to HIV-infected humans and that viral loads can be abated to levels below the assay detection limit by adjusting the drug dosages and boosting procedures.

### A highly sensitive viral load detection assay shows profound suppression of viral replication by H-iART

To check the presence of low-level viremia in SIVmac251-infected macaques under H-iART, we lowered the detection limit to 3 copies of viral RNA/mL and re-measured viral loads in some selected pooled serum samples. We found no evidence for low-level viral replication in plasma of all of the macaques tested (Table 1). Of note, viral RNA was below the assay detection limit in the plasma samples taken from macaque 4887 before its last viremic episode, supporting the hypothesis that H-iART was able to completely control viral replication in the periphery, despite the presence of a major CNS reservoir (Fig. 4F). Analyses conducted on lymph node biopsies (inguinal) showed that four out of six macaques analyzed had levels of cell-associated RNA below the limit of detection of the assay (*i.e.* 2 copies/5*10^5^ cells/mL) (Table 2). The presence of cell-associated RNA in lymph nodes was independent of baseline viremia at treatment initiation (*Logit* analysis *P* = 0.801), thus supporting the idea that the suppressive efficacy of H-iART is not confined only to those macaques starting from moderate viral loads. In addition, cell associated RNA measured in samples taken from rectal biopsies was below the assay detection limit in all animals analyzed, supporting the idea of full suppression of peripheral viral replication (Table 2). This was rather surprising, because other antiretroviral regimens adopted in macaques proved unable to completely control viral RNA in anatomical sanctuaries [3], [24].

**Table 2:** Cell associated RNA and DNA in lymph nodes and rectum.

Macaque	Time (days)	RNA		DNA	
		Lymph Node (inguinal)	Rectum	Lymp Node (inguinal)	Rectum
**P157**	289 (see Fig. 2)	<2	<2	<2	<2
**P177**	289 (see Fig. 2)	<2	<2	<2	<2
**P185**	289 (see Fig. 2)	<2	<2	<2	<2
**P188**	289 (see Fig. 2)	N/A	<2	N/A	<2
**BD12**	140 (see Fig. 3)	N/A	<2	N/A	<2
**4416**	315 (see Fig. 4)	N/A	<2	N/A	<2
**BD53**	140 (see Fig. 3)	N/A	<2	N/A	<2
**BD64**	484 (see Fig. 4)	136	<2	<2	<2
**BD69**	472 (see Fig. 4)	11	<2	7	<2
**4887**	484 (see Fig. 4)	<2	<2	6	<2

The limit of detection for both RNA and DNA assays is 2 copies/5*10^5^ cells. Each assay was conducted in triplicate. The time points selected for the analyses are shown as days from the zero point adopted in figures 2, 3 and 4.

### H-iART impacts on viral DNA in PBMCs, lymph nodes and rectum

**Figure 5:**
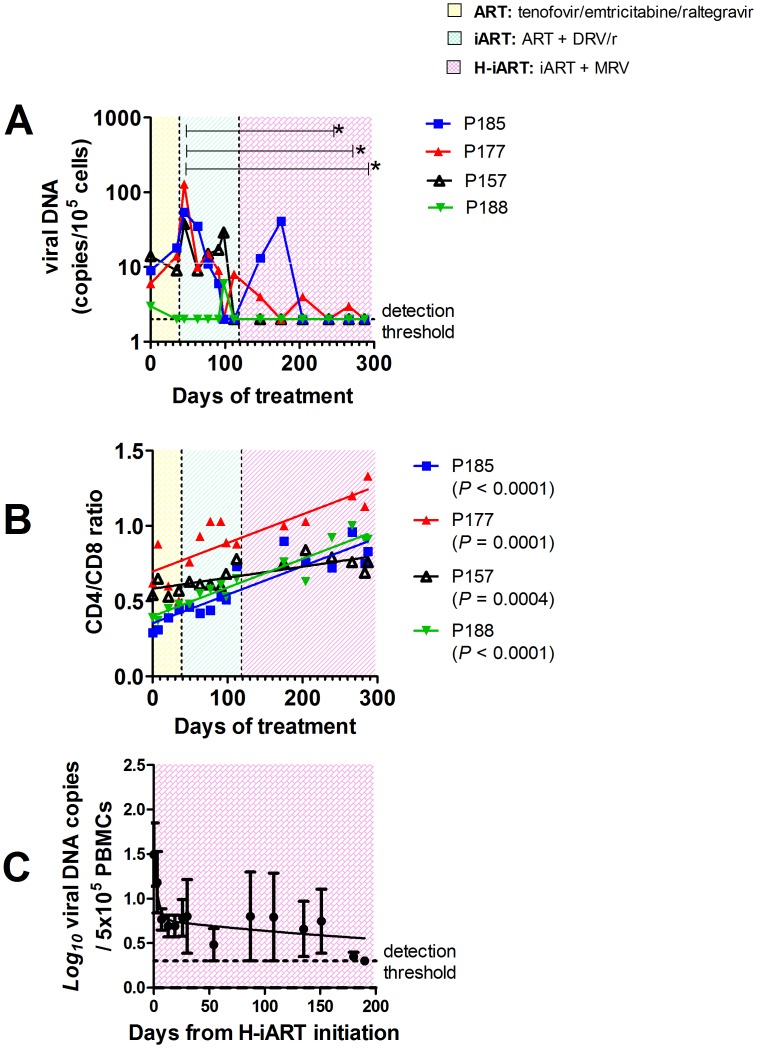
H-iART decreases viral DNA in PBMCs and increases the CD4/CD8 ratio. Panel A: Viral DNA in PBMCs. Panel B: CD4/CD8 ratios. Both panels show the results from macaques enrolled in the pilot study. The sequential treatments are represented by the colored areas. In panel A, asterisks mark the significant differences from baseline values (*P*<0.05), as detected by Bonferroni's test. Panel C: Three-phase decay dynamics of total viral DNA of three macaques (BD69, 4416, P255) to which all H-iART drugs were administered simultaneously and for which viral DNA values from treatment initiation were available. Each time point represents average values (± SEM).

In the pilot study presented above, we unexpectedly found that H-iART profoundly impacted on viral DNA. First, there was a late viral DNA decay to levels below the assay detection limit which was associated with the addition of MRV to the drug cocktail (Fig. 5A). In addition, the CD4/CD8 ratio, the decrease of which is a marker of the viral reservoir and/or ongoing viral replication [7], [8], significantly increased during treatment (Fig. 5B). Of note, viral DNA in PBMCs also fell below the assay detection limit in all macaques included in the group treated with H-iART *ab-initio* (median treatment duration = 125 days, range from 45 to 174 days), *i.e.* no viral DNA copies were detectable in six out of six repeats with a threshold sensitivity of 2 copies/5*10^5^ cells.

Moreover, we could not detect viral DNA in lymph node and rectal tissue biopsies (detection limit: 2 copies/5*10^5^ cells, three repeats per sample) in all the macaques of the pilot study tested (Table 2). Lymph node viral DNA was also below the assay detection limit in one of three macaques from those treated with H-iART *ab-initio*, while viral DNA was below the limit of detection in rectal biopsies of all the macaques of the same group (Table 2). The results were further validated by excluding the presence of PCR inhibitors using spiked DNA for selected samples (Table S2).

### Viral DNA decay dynamics during H-iART

The dynamics of the viral DNA decay during H-iART were studied in those animals to which all H-iART drugs were administered simultaneously and for which viral DNA measurements were available.

The levels of viral DNA in PBMCs during time were consistent with a three-phase decay, with the first two phases paralleling the two-phase decay of viremia, and a third, slower phase occurring after viremia had fallen to levels below the assay detection limit (Fig. 5C). This last phase of the viral decay has been ascribed to the latently infected T-cell numbers [18]. This result was noteworthy, because no such decreasing trends in viral DNA had been observed in animals treated with iART (*i.e.* without MRV) [7].

### H-iART impacts on the memory T-cell pool

**Figure 6:**
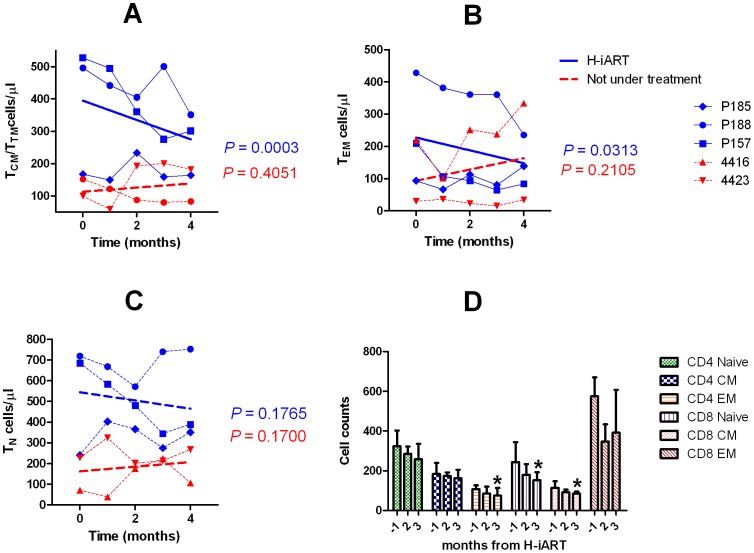
T-cell subpopulation dynamics during H-iART. Panel A: CD4^+^ central and transitional memory T-cells (T_CM_/T_TM_) Panel B: CD4^+^ effector memory T-cells (TEM). Panel C: CD4^+^ naïve T-cells (TN). Panel D: HLA-DR^+^ T-cell subsets. In panels A–C, individual data points are presented for each animal. The significantly decreasing trends are shown by the solid regression lines. Dashed lines refer to non significant trends (*P*>0.05). In panel D, data are presented as means ± SEM from three animals and significantly decreasing trends are shown by the asterisks.

In line with the reportedly stimulating effect of the major CCR5 ligand RANTES on T-cell proliferation [25] some studies suggested that MRV, by acting as an antagonist of this cytokine, might alter the T-cell dynamics *in vivo* [26]. To study these phenomena, the CD4^+^ T-cell subpopulations were analyzed by six-color flow-cytometry at different time points following addition of MRV to the therapeutic regimen (Fig. 6). To avoid biasing the result with the possible effects of a detectable viral load on the T-cell subpopulations, these tests were conducted on PBMCs from macaques P157, P185 and P188 which already displayed a viral load below the assay detection limit when MRV was added (Fig. 2). Results showed that H-iART decreased the memory CD4^+^ T-cell numbers over time (Fig. 6A,B), while it carried out no significant effect on the naïve T-cell subpopulation (Fig. 6C). This result is in accordance with the *in-vitro* inhibitory effect of MRV on the proliferation of sorted memory T-cell subpopulations (Fig. S7). MRV significantly decreased the numbers of activated (HLA-DR^+^) CD4^+^ T_EM_ cells (Fig. 6D). This effect is in line with decreased levels of immune activation already observed in humans treated with this drug [26], [27]. In conclusion, MRV decreased the number of memory T-cells as well as T_EM_ cell-activation. Since these two parameters are linked to the magnitude of the viral reservoir and ongoing viral replication [9], [28], this effect is in good agreement with the aforementioned three-phase decay of viral DNA induced by MRV (Fig. 5C).

### MRV impacts on the viral set point following therapy suspension

**Figure 7:**
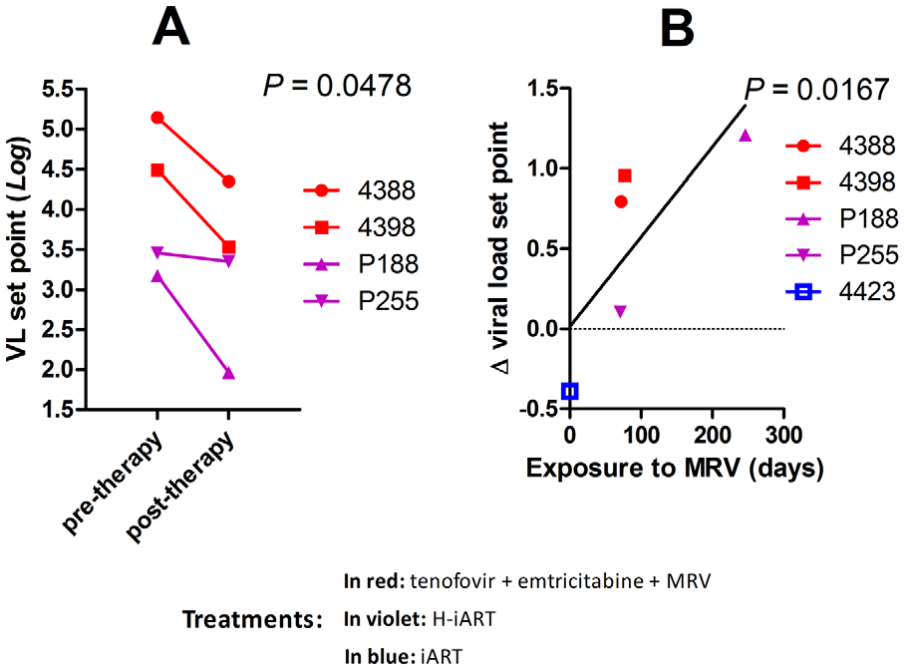
MRV decreases the post-therapy viral load set point. Panel A: Pre and post therapy *Log_10_* viral load set points of four SIVmac251 infected macaques treated with MRV-containing therapies. The *P*-value shown is the result of paired *t*-test analysis. Panel B: Correlation between the *Log_10_* Δ viral load set point (*i.e.* the difference between pre and post therapy viral load set points) and time of exposure to MRV. Correlation was investigated using Pearson's coefficients. The treatment of macaques 4388 and 4398 prior to therapy suspension is shown in Figure S7 and Text S2.

The results so far obtained were in line with a recently issued report which suggested that MRV decreased the magnitude of the viral reservoir in HIV-1-infected individuals [26]. This study, which was unable to provide conclusive evidence, did not show an impact of MRV on the viral set point following therapy suspension, a parameter stringently associated with the extent of the viral reservoir [7], [29], [30]. To test this hypothesis, we analyzed the difference in the pre and post-therapy viral set points in those macaques from our cohort that had received MRV and that had undergone therapy suspension (for treatment details see Figs. 2, 4 and Text S3). Results show that treatment with MRV is associated with a reduction of the viral set point post-therapy (Fig. 7A), and that the extent in the viral set point decrease depends on the total exposure to the drug (Fig. 7B). These results are suggestive of an independent effect of MRV on the viral set point following therapy suspension and add credit to the hypothesis that MRV may contribute to an anti-reservoir effect of H-iART.

### H-iART improves the spontaneous control of viral load following a previous anti-reservoir strategy

**Figure 8:**
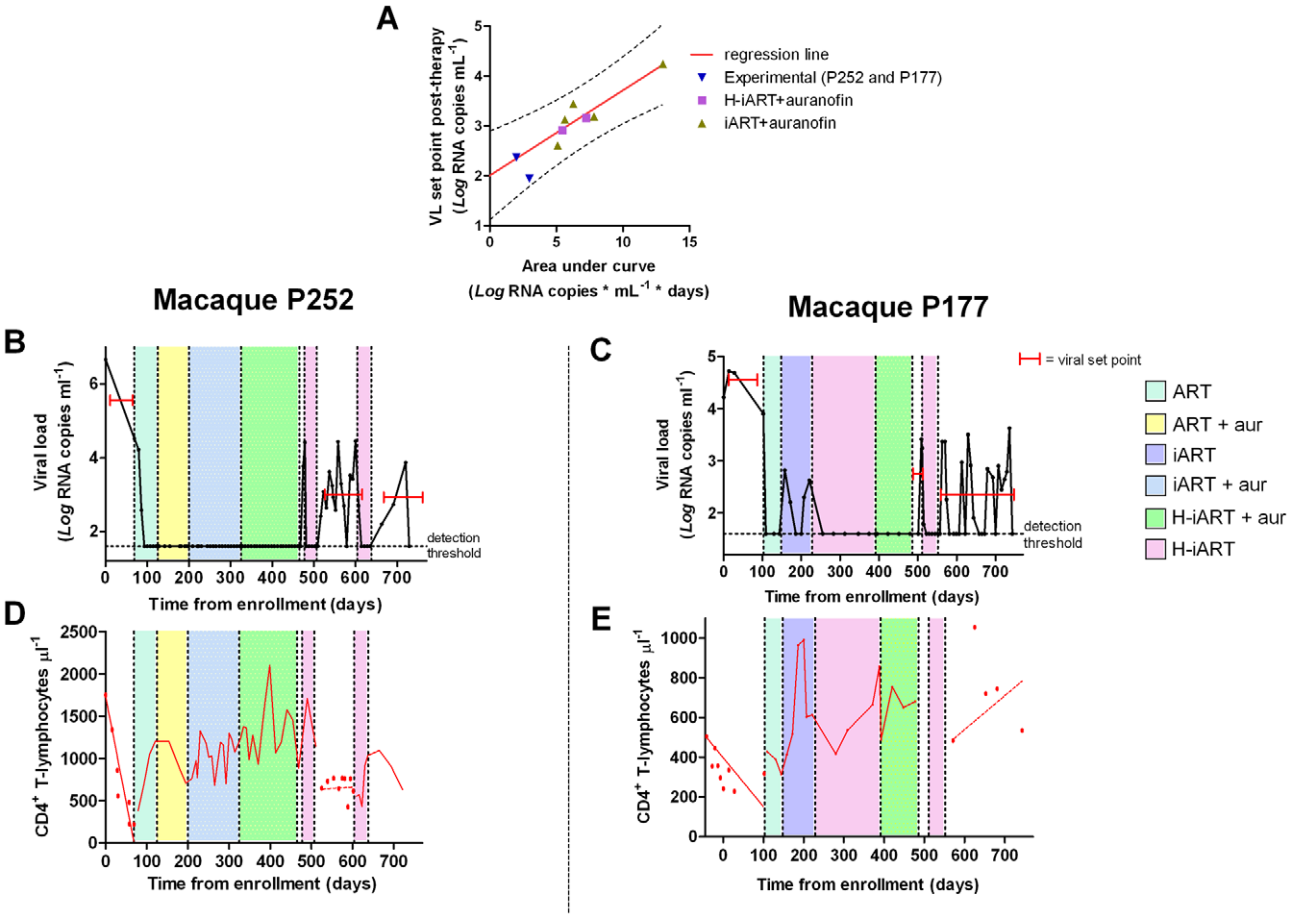
A short cycle of H-iART at viral rebound after structured treatment interruption improves the effects of auranofin-based anti-reservoir therapies on the eventual viral load set point. Panel A: Correlation between the area under the viral load curve at peak (AUC) following viral rebound and the eventual viral load set point. Panels B,C: Viral loads from infection of macaques subjected to the combined antireservoir/antiretroviral treatment protocol (see main text). The red bars mark the viral set points (calculated as the mean of the available *Log_10_* viral load measurements). Panels D,E: CD4 counts. The values before and after the treatment periods are shown by the individual data points, and trends are described by the regression lines (solid: significant slopes; dashed: non significant slopes).

Finally, given the aforementioned effects of H-iART, we tested whether this therapeutic regimen might be adopted to improve the effect of a previous anti-reservoir strategy based on the anti-memory drug auranofin in combination with antiretrovirals [7]. Upon interruption of this anti-reservoir treatment, SIVmac251-infected macaques experience an acute infection-like condition, *i.e.* an initial viral load peak followed by rapid containment of viral load [7]. The peak, which is rapidly reached upon virus re-appearance in plasma, is associated with the reconstitution of the viral reservoir, as shown by the previously published independent association between the area under the curve (AUC) describing the initial peak of viral load and the eventual viral load set point ([7] see also Fig. 8A). From this association, it follows that decreasing the AUC at peak artificially through a cycle of H-iART should limit the reconstitution of the viral reservoir and may result in spontaneous control of viral load following H-iART suspension.

The experiment was attempted in two macaques. A first macaque (P252) was treated with a one-month cycle of H-iART at viral load rebound, after the suspension of the aforementioned auranofin/antiretroviral regimen. Another macaque (P177) was treated with auranofin in addition to H-iART as a follow-up of the treatment presented in the pilot study. Eventually, following therapy suspension, P177 was subjected to a short H-iART cycle at viral rebound, similar to that administered to P252. In both cases, the short H-iART cycle promptly abated viral load to levels below the assay detection limit, thus efficiently decreasing the initial AUC (Fig. 8 A–C).

The macaques showed exceptionally low viral set points after the short cycle of H-iART was suspended, in line with the expected values calculated on the basis of our AUC/viral set point correlation curve (Fig. 8A). Both macaques periodically displayed viral load peaks that subsequently decreased to low-level viremia (<500 copies of viral RNA/mL) or to levels below the assay detection limits. The CD4 slope was non-significant during the follow-up period (*P* = 0.7079 for P252 and *P* = 0.2319 for P177; Fig. 8D,E), in line with the previous observation that the CD4 slope following therapy suspension identifies the impact of a treatment on the viral reservoir [7]. Conversely, CD4 counts had shown significantly decreasing trends in both macaques before all treatments were started (*P*<0.0001 for P252 and *P* = 0.0039 for P177; Fig. 8D,E), thus supporting the concept that the therapies adopted significantly impacted on the natural course of the disease.

Consistently with its exceptional reduction of the AUC at peak, macaque P177 showed a remarkable degree of spontaneous control of viral load during six months of follow-up, which was not yet considerable as, but seemingly close to a drug-free remission of the disease (Fig. 8C). In this macaque, viral load was maintained at levels below the assay detection limit during the periods between peaks (detection limit: 40 RNA copies/mL) and, when the RNA detection limit was further lowered to 3 copies/mL, no evidence of residual viremia was found (see Table 1). This control of viral replication could hardly be ascribed to cell-mediated responses, in that a moderate increase in the number of IFN-γ positive spots could be detected only at viral rebound but not during the viral set point (Fig. S8), thus suggesting that H-iART induced a true containment of the viral reservoir reconstitution, similarly to other experimental strategies restricting the formation of the viral reservoir during acute infection [29], [30], [31]. We conclude that a short course of H-iART, in line with the highly suppressive effect of this therapeutic regimen on SIVmac251, may prevent the viral reservoir reconstitution following suspension of a previous anti-reservoir therapy and result in a drug-free spontaneous control of viral load.

## Discussion

Some investigators recently questioned the robustness of primate models, citing the difficulty of obtaining, with the cross-active drug options available, full viral suppression in sanctuaries and viral loads below the assay detection limits for prolonged periods [32], [33]. The results reported in the present article do not support this argument.

1) Since a good animal model should mirror full viral suppression in humans, we checked viral loads in plasma for prolonged periods and analyzed the presence of viral nucleic acids in anatomical sanctuaries. The level of abatement of viral nucleic acids that we found in the present study in peripheral blood and anatomical sanctuaries of the majority of the macaques tested provide the maximum degree of viral suppression so far observed in antiretroviral treated primates. The level of reproducibility of these results is shown by the fact that they were obtained in a heterogeneous group of macaques, likely mirroring a wide number of possible disease conditions in humans. This is the first report, to our knowledge, of a therapy capable of stably controlling viral replication to levels below the assay detection limits also in macaques in the advanced stage of the disease, since the studies so far published have been able to report control of SIV replication only during acute infection [12] or in the early chronic phase of the disease [3]–[6]. Apart from mimicking the clinical conditions of a significant portion of HIV-infected individuals who are diagnosed in the chronic or pre-AIDS stages of the disease, this ‘late’ treatment allows excluding those macaques able to spontaneously control the infection, a phenomenon which usually occurs soon after the acute infection phase [34]. For the macaques enrolled in this study, the average plasma viral load at the time of therapy initiation was of 4.8±1.1 *Log_10_* RNA copies/mL (mean ± SD). This value is lower than those reported in some articles during chronic SIVmac infection of macaques [35], [36], but similar to those published in other articles [37], [38]. As in this study we have not included macaques with viral loads during chronic infection higher than 6.8 *Log_10_* RNA copies/mL or with the rapid progressor phenotype, the effect of our H-iART regimen on this more aggressive course of SIV infections remain to be ascertained.

Of note, persistence of the virus at low level in the lymph nodes of a minority of H-iART treated macaques provides another similarity of our macaque model with clinical conditions observed in humans infected with HIV-1, as this anatomical sanctuary has recently been shown to be a major site for ongoing viral replication in humans [39]. Studies of drug penetration in this anatomical compartment will be necessary to overcome this limitation in both macaques and humans.

**Figure 9:**
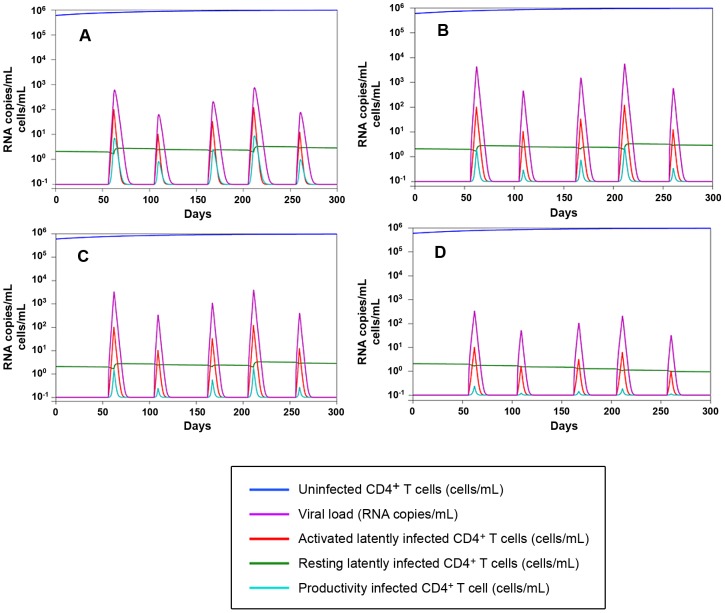
Numerical simulations of the Rong and Perelson model with programmed expansion and contraction of the viral reservoir. Panel A: Simulation of the viral load and viral reservoir dynamics in a human model. The 300 days simulation is based on the five differential equations model (4) in [40], where burst size is assumed to be 2000 RNA copies/day. The peaks in the viral load (violet) correspond to the periods of activation of latently infected CD4^+^ T-cells. Proliferation rate and drug efficacy are assumed to be respectively 1.4 and 0.85. For starting data see Table S3. Panels B–D: Simulation of the viral load and viral reservoir dynamics infection in a macaque model. According to [41] burst size is assumed to be 55000 RNA copies/day, which determines higher peaks in the viral load than in the previous human model. Panel B: proliferation rate = 1.4, drug efficacy = 0.95. Panel C: proliferation rate = 1.4, drug efficacy = 0.99. Panel D**:** proliferation rate = 0.945, drug efficacy = 0.99. The activation function adopted to simulate lymphocyte encounter with antigens is illustrated in Fig. S9 (for further detail, see Text S1).

2) As in any well respected science, the results are in good agreement with mathematical models (Fig. 9), and are mathematically predictable (as an example, see Fig. 8A). In this regard, important insight into the necessity for a multidrug regimen to control viral loads in macaques can be derived from a mathematical model developed by Rong and Perelson [40] and based on experimental observations [8]. This model suggests that a superior drug efficacy is required in simian AIDS to control viral replication (Fig. 9) because of the viral burst size, (*i.e.* the average number of virions produced by a single productively infected cell in a day). The viral burst size was shown to be higher in SIV infection as compared to HIV-1 infection [41], where a lower drug efficacy is expected to be sufficient to maintain viral control (Fig. 9A–C). Also a drug acting on the proliferation rate of activated T-cells, such as MRV (which antagonizes the proliferative effect of RANTES, see ref 25 and Fig. S7), appears to be important for containment of the viral blips (Fig. 9D). These simulations also show that the decreased proliferation rates may impact on the viral reservoir size (half-life: ≈200 days, see Text S4, S3, S2, S1 and Fig. 9D), which shows a half-life of the same order of magnitude as that calculated by analyzing the dynamics of the viral DNA decay during H-iART (Fig. 5).

3) According to the idea that a good animal model should represent a vanguard for future treatments to be tested in humans, our quest for increased drug efficacy in the macaque AIDS model allowed identifying unexpected benefits of H-iART on the immune system. Apart from the possible impact of H-iART on the viral reservoir (a concept supported by recent data in humans [42]), reduction by MRV of the memory T-cell subpopulation may restrict one major source for viral spread and ongoing viral replication. A decrease in the memory T-cell size is a logical expectation of the anti-proliferative effect exerted by MRV through CCR5 inhibition (Fig. S7), as antigen-driven proliferation contributes to maintenance of the size of this T-cell subpopulation [8]. It is well known that memory T-cells are a preferential target of HIV-1 replication [43], and that their decrease may affect the overall viral dynamics *in vivo*. In this regard, the MRV-induced decrease in the memory T-cell size is not only unlikely to be dangerous but, rather, likely to be beneficial. This hypothesis is supported by results showing that the pool of T_CM_ cells is a correlate of anergy towards the viral antigens in *Macaca mulatta* but not in *Cercocebus atys*, which is naturally resistant to CD4_+_ T-cell loss and full-blown AIDS [44]. In addition, the results obtained with the present macaque model suggest that a short cycle of H-iART could be used for improving the efficacy of our previous anti-reservoir treatment based on auranofin and strengthen the idea that an arrest in disease progression may be obtained during the chronic phase of the disease. Although the data on the combined effect of the two subsequent treatment cycles are derived from a limited number of macaques, the result obtained is corroborated by the fact that no similar trend was observed in the same animals prior to starting therapy [5], [7] or in historical controls that had not received H-iART at rebound [7]. Of note, although certain major histocompatibility complex (MHC) class I alleles, including Mamu-A*01 and Mamu-B17* are associated with slow disease progression in SIV infected macaques [45], [46], independently, the presence of these alleles is not predictive for disease outcome [47], and none of our macaques presented the protective alleles in association (Table S1). Instead, P177, which, following our therapies, remarkably controlled viral load, presented the HLA Mamu-B*01 allele, that is associated with aggressive simian lentivirus infection [48]. In line with this genotype, P177 showed a significant immune deterioration before our treatments were initiated (Fig. 8C).

Finally, recent analyses [reviewed in 49] re-evaluated the necessity of wide numbers of subjects as a support for breakthrough findings, such as, in this case, the obtainment of a condition close to a persistent suppression of viremia in the absence of ART.

If the results of the present study should prove reproducible in humans, H-iART could represent a useful tool for improving the viro-immunological conditions of HIV-infected individuals and a useful addition to experimental anti-reservoir strategies.

## Supporting Information

Figure S1 **Variability of the quantitative real-time RT-PCR assay for measurement of viral RNA.** Panel A: Standard curves run on three different occasions. Panel B: Coefficients of variation at different starting RNA concentrations. The coefficients of variation were calculated as the standard deviation of each group of values (starting from the same RNA concentration) divided by the mean value and multiplied by 100. Lack of concentration-dependence shows that the variability at the different concentrations is due to random fluctuations rather than to loss of sensitivity at the extremes of the curve.
(TIF)

Figure S2 **Structural analysis of SIVmac251 susceptibility to darunavir.** Panel A: Sequence alignment of the protease of HIV-1 subtype B [PDB: 2HS1,V32I Mutant], HIV-2 [PDB: 3ECG], and SIVmac251 [PDB: 2SAM]. The sequence alignment is based on a structural alignment performed using the VAST algorithm. Regions showing significant structural alignment are presented in blue, with the highly conserved residues shown in red. The mutations found in HIV-1 infected individuals failing DRV-based drug regimens are highlighted above the alignments (the green arrows indicate the primary resistance mutations; black arrows indicate secondary resistance mutations). Panel B: Comparison between the DRV/HIV-1-protease experimental model (green sticks) and our DRV/SIVmac251-protease theoretical model (cyan transparent sticks). Yellow dashes depict the hydrogen bonds and the red sphere indicates the position of the structural water molecule involved in drug-protein interactions. Amino acids and DRV are represented in CPK. The methodology adopted for the molecular modeling, is described in detail in the Text S1.
(TIF)

Figure S3 **Viral loads of SIVmac251-infected macaques before and during treatment with maraviroc, tenofovir and emtricitabine.** Asterisks show the significant differences between values at start of follow-up and during treatment [*P*<0.05, Bonferroni's post test following significant (*P*<0.05) repeated-measures ANOVA].
(TIF)

Figure S4 **CD4^+^ T-cell counts of six uninfected and four SIVmac251 infected macaques.** Individual data points, as well as means (± SEM), are shown for each group.
(TIF)

Figure S5 **Examples of SIVmac251 infection course in rhesus macaques.** Depicted is the progression of viremia in a cohort of seven SIVmac251 infected rhesus macaques. In red are the macaques displaying similar viral loads as those of the animals enrolled in the pilot study (see text).
(TIF)

Figure S6 **Treatment with H-iART recovers CD4^+^ T-cell counts decreased by pathogenic SIVmac251 infection.** Five macaques are considered for which pre-infection and pre-treatment CD4^+^ T-cell counts were available. Values during H-iART refer to a median period of 89 days (range: 83–89 days). Data have been analyzed using one-way ANOVA followed by Newmann-Keuls test.
(TIF)

Figure S7 **Maraviroc decreases T-cell proliferation** ***in vitro*** **.** The percentage inhibition of proliferation induced by 0.1 µM MRV in CD4^+^ T-cells activated with αCD3/αCD28 is shown. Data are shown as mean + SEM and are derived from two experiments. CM: central memory; TM: transitional memory; EM: effector memory. *P* = 0.0417; Friedman's test.
(TIF)

Figure S8 ELISPOT analysis of the number of interferon-γ secreting cells/1.5 * 10^5^ PBMCs. The analyses refers to A: macaque P252 and B: macaque P177. The time points selected are shown as days from the zero point adopted in figure 8 of the main text.
(TIF)

Figure S9 **Activation function.** The step function *f*(*t*) which determines the activation of resting latently infected CD4^+^ T-cells. Times between two activation periods follow a Poisson distribution with a mean of 50 days. The length of activation periods follows a uniform distribution over an interval of 4 to 6 days.
(TIF)

Table S1 **Viro-immunological background and therapeutic regimens of the SIVmac251 infected macaques employed in the study.** MHC alleles analyzed are the following: Mamu A*01; A*02; A*08; A*11; B*01; B*03; B*04; B*08; B*17. The CD4 nadir is chosen as the lowest pre-therapy T-CD4 count available. Therapeutic regimens described in the present article are highlighted in violet.
(TIF)

Table S2 **Validation of the real-time PCR assay for SIVmac251 DNA quantification in PBMCs and lymph node biopsies.** The limit of detection of the assay is 2 copies/5*10^5^ cells. As a control of the assay variability and to exclude PCR inhibition, spiked DNA measurements for each sample were used.
(TIF)

Table S3 **Starting data for the numerical simulations of the viral load and reservoir dynamics.**
(TIF)

Text S1 **Mathematical modeling and numerical simulations.**
(DOCX)

Text S2 **Bioinformatic analyses and molecular modeling studies.**
(DOCX)

Text S3 **Retrospective analysis of the response of SIV-mac251 to maraviroc** ***in vivo*** **.**
(DOCX)

Text S4 ***In vitro* measurement of the effect of MRV on T-cell proliferation.**
(DOCX)
