# Supplementary figures and images for: A Highly Intensified ART Regimen Induces Long-Term Viral Suppression and Restriction of the Viral Reservoir in a Simian AIDS Model
Source: PLoS Pathog. 2012 Jun 21;8(6):e1002774. doi: 10.1371/journal.ppat.1002774 (PMC3380955; doi:10.1371/journal.ppat.1002774)

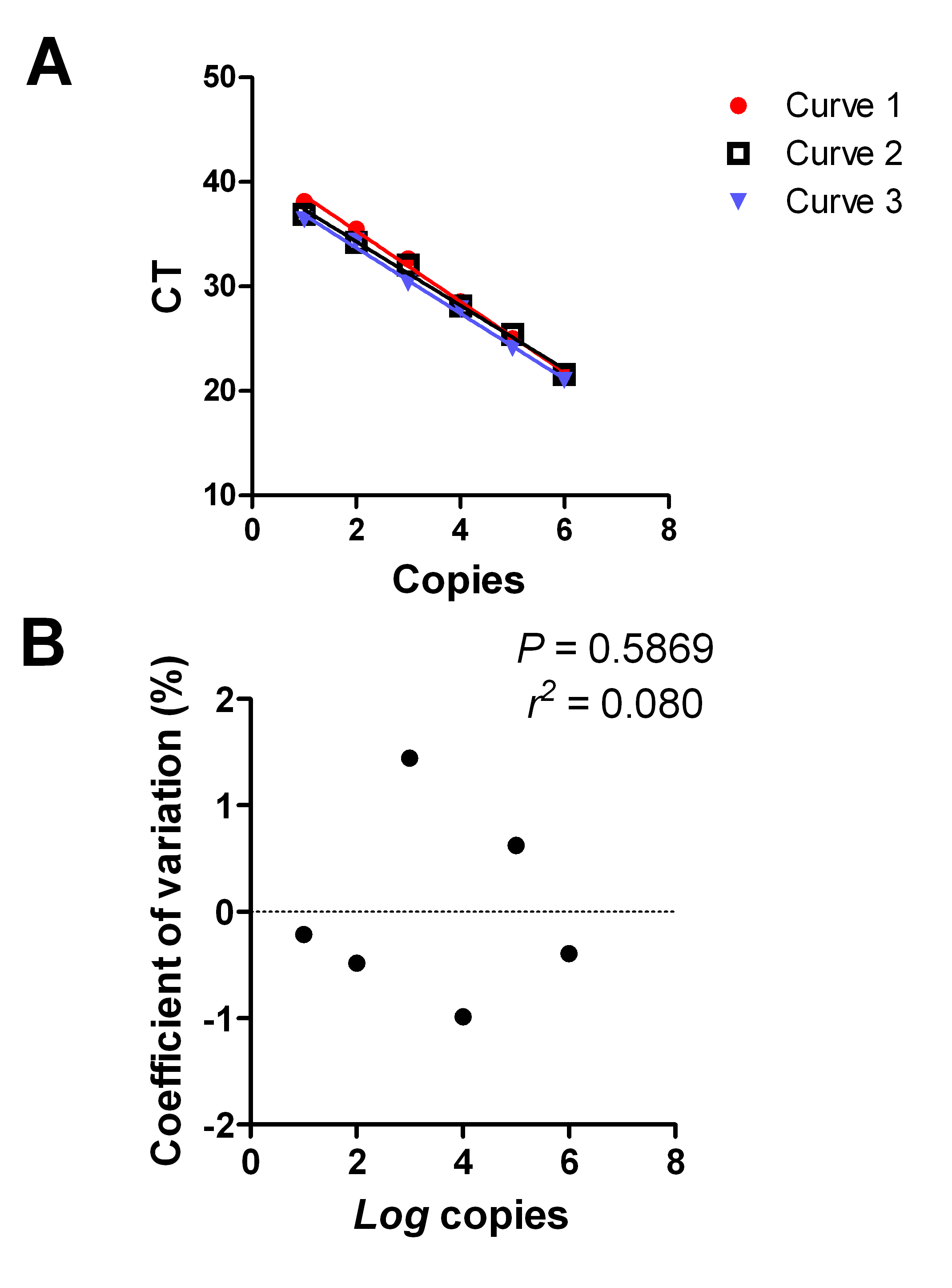

Supplement: Figure S1 — Variability of the quantitative real-time RT-PCR assay for measurement of viral RNA. Panel A: Standard curves run on three different occasions. Panel B: Coefficients of variation at different starting RNA concentrations. The coefficients of variation were calculated as the standard deviation of each group of values (starting from the same RNA concentration) divided by the mean value and multiplied by 100. Lack of concentration-dependence shows that the variability at the different concentrations is due to random fluctuations rather than to loss of sensitivity at the extremes of the curve. (TIF) [file ppat.1002774.s001.tif]

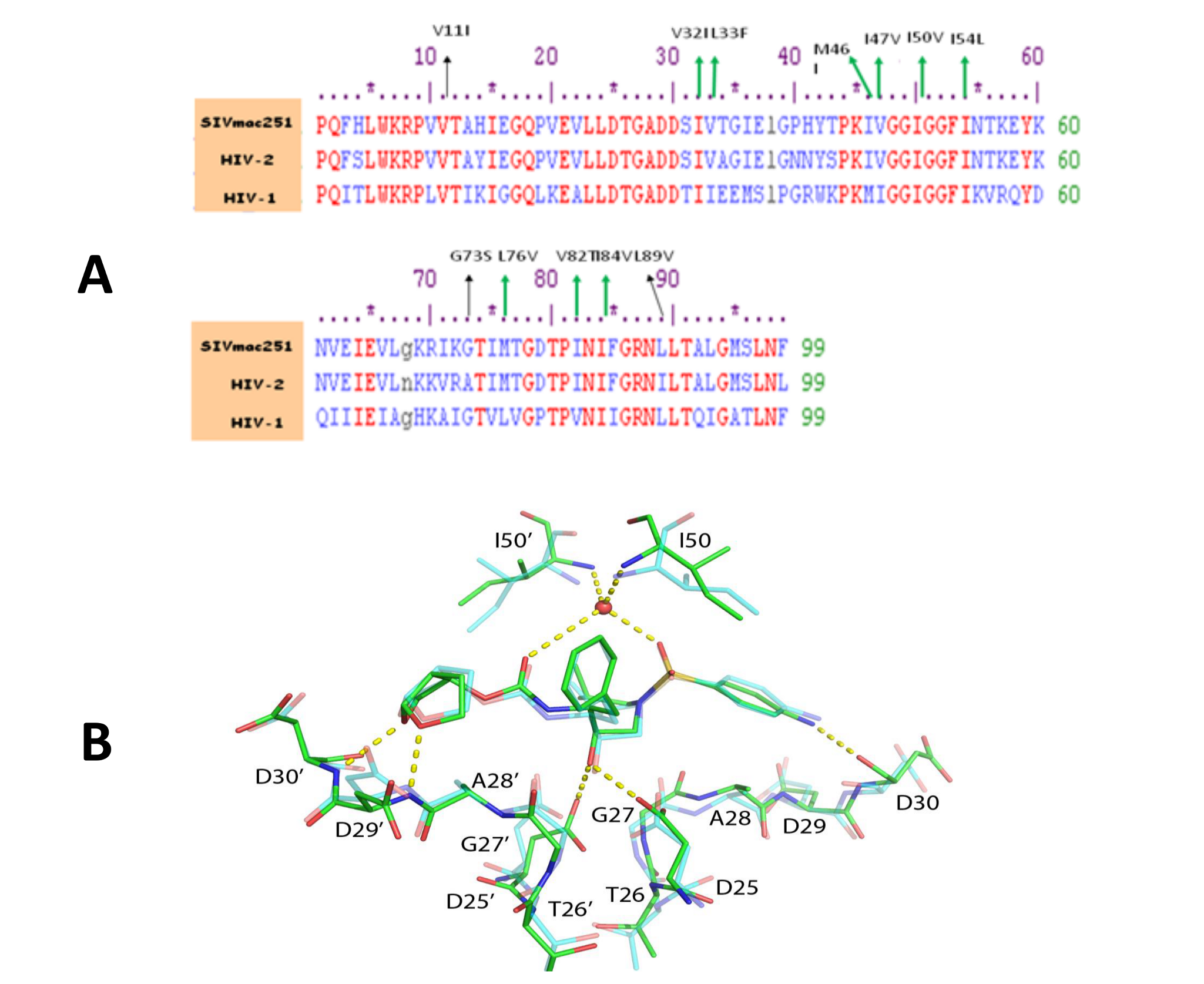

Supplement: Figure S2 — Structural analysis of SIVmac251 susceptibility to darunavir. Panel A: Sequence alignment of the protease of HIV-1 subtype B [PDB: 2HS1,V32I Mutant], HIV-2 [PDB: 3ECG], and SIVmac251 [PDB: 2SAM]. The sequence alignment is based on a structural alignment performed using the VAST algorithm. Regions showing significant structural alignment are presented in blue, with the highly conserved residues shown in red. The mutations found in HIV-1 infected individuals failing DRV-based drug regimens are highlighted above the alignments (the green arrows indicate the primary resistance mutations; black arrows indicate secondary resistance mutations). Panel B: Comparison between the DRV/HIV-1-protease experimental model (green sticks) and our DRV/SIVmac251-protease theoretical model (cyan transparent sticks). Yellow dashes depict the hydrogen bonds and the red sphere indicates the position of the structural water molecule involved in drug-protein interactions. Amino acids and DRV are represented in CPK. The methodology adopted for the molecular modeling, is described in detail in the Text S1. (TIF) [file ppat.1002774.s002.tif]

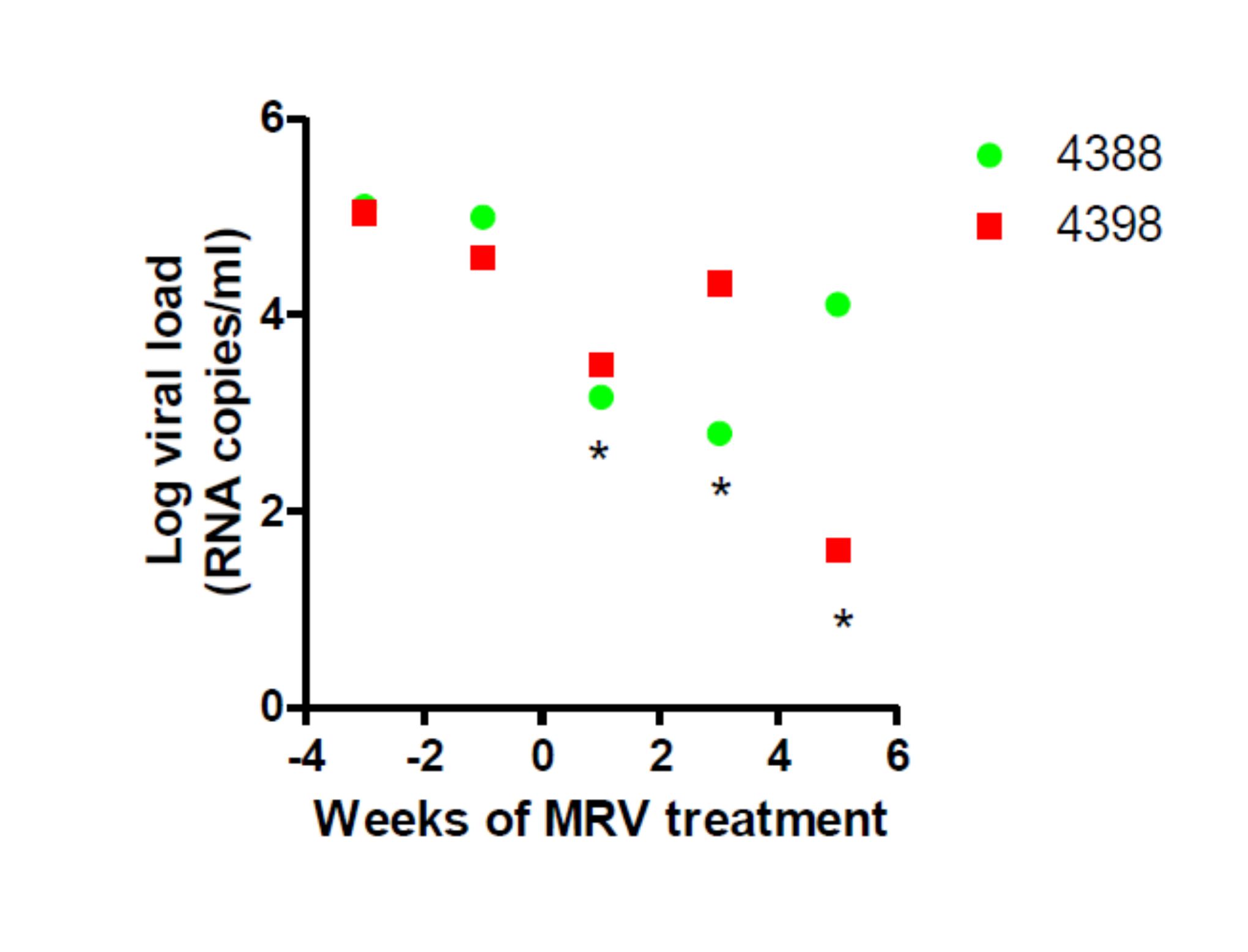

Supplement: Figure S3 — Viral loads of SIVmac251-infected macaques before and during treatment with maraviroc, tenofovir and emtricitabine. Asterisks show the significant differences between values at start of follow-up and during treatment [P<0.05, Bonferroni's post test following significant (P<0.05) repeated-measures ANOVA]. (TIF) [file ppat.1002774.s003.tif]

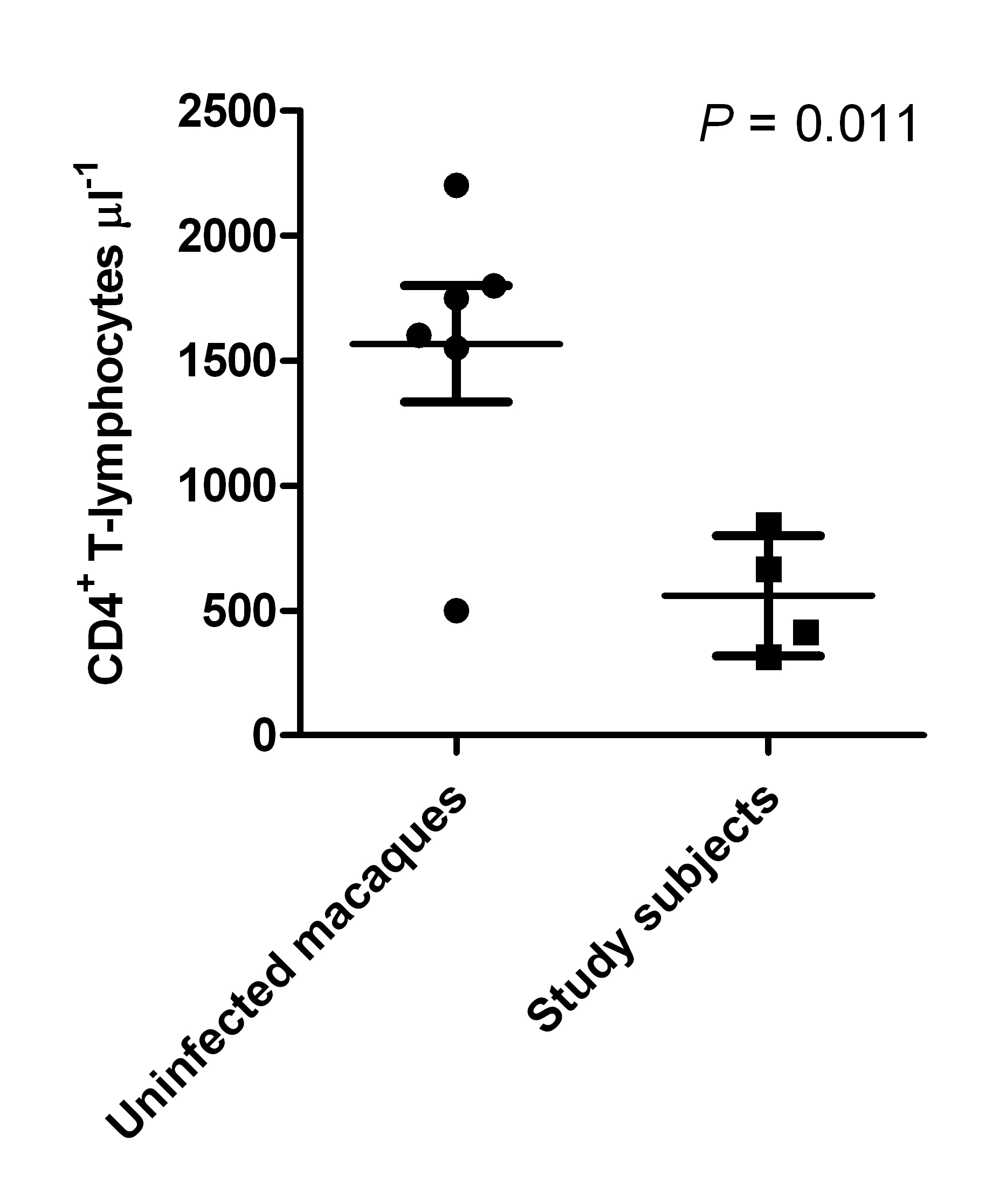

Supplement: Figure S4 — CD4+ T-cell counts of six uninfected and four SIVmac251 infected macaques. Individual data points, as well as means (± SEM), are shown for each group. (TIF) [file ppat.1002774.s004.tif]

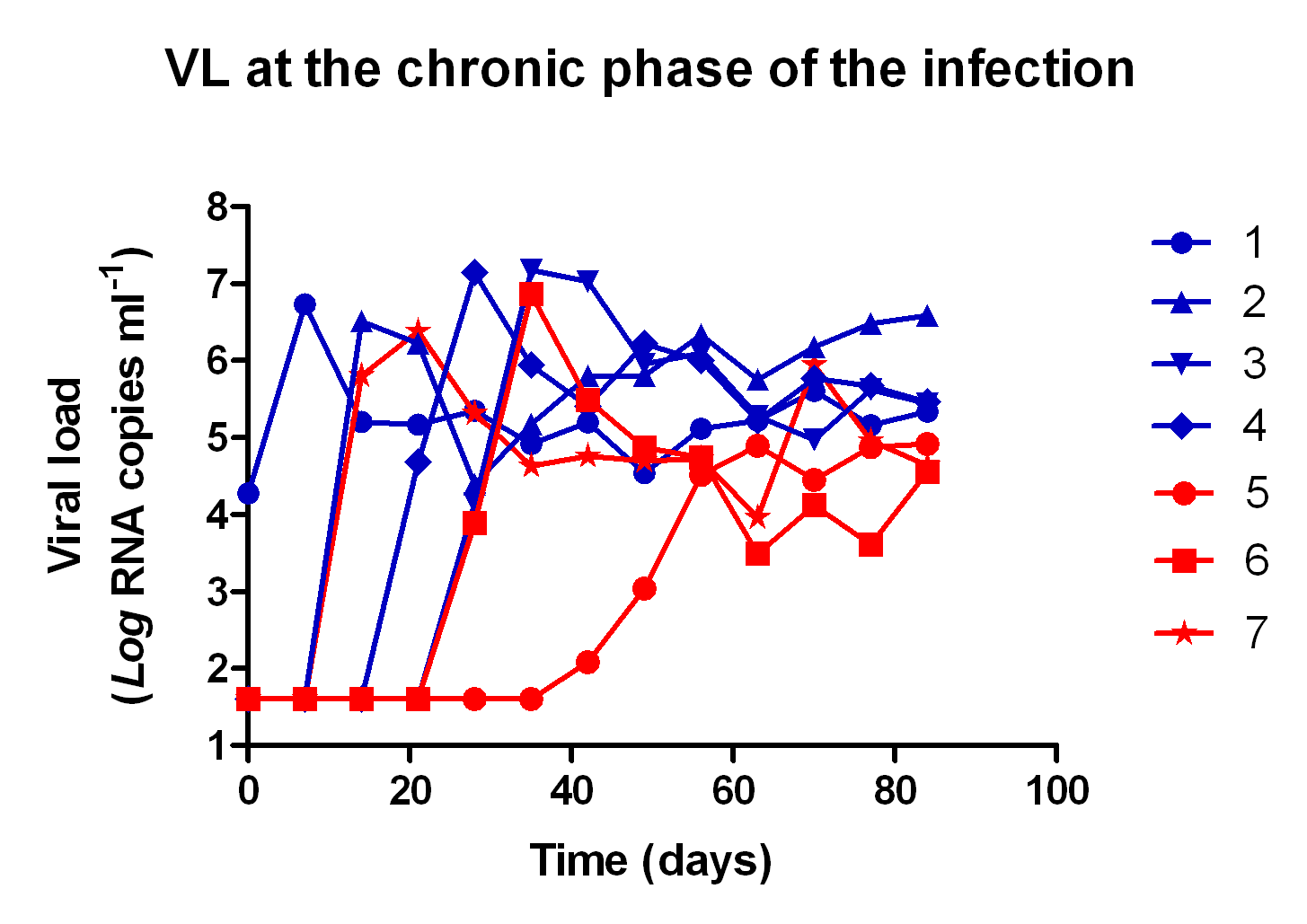

Supplement: Figure S5 — Examples of SIVmac251 infection course in rhesus macaques. Depicted is the progression of viremia in a cohort of seven SIVmac251 infected rhesus macaques. In red are the macaques displaying similar viral loads as those of the animals enrolled in the pilot study (see text). (TIF) [file ppat.1002774.s005.tif]

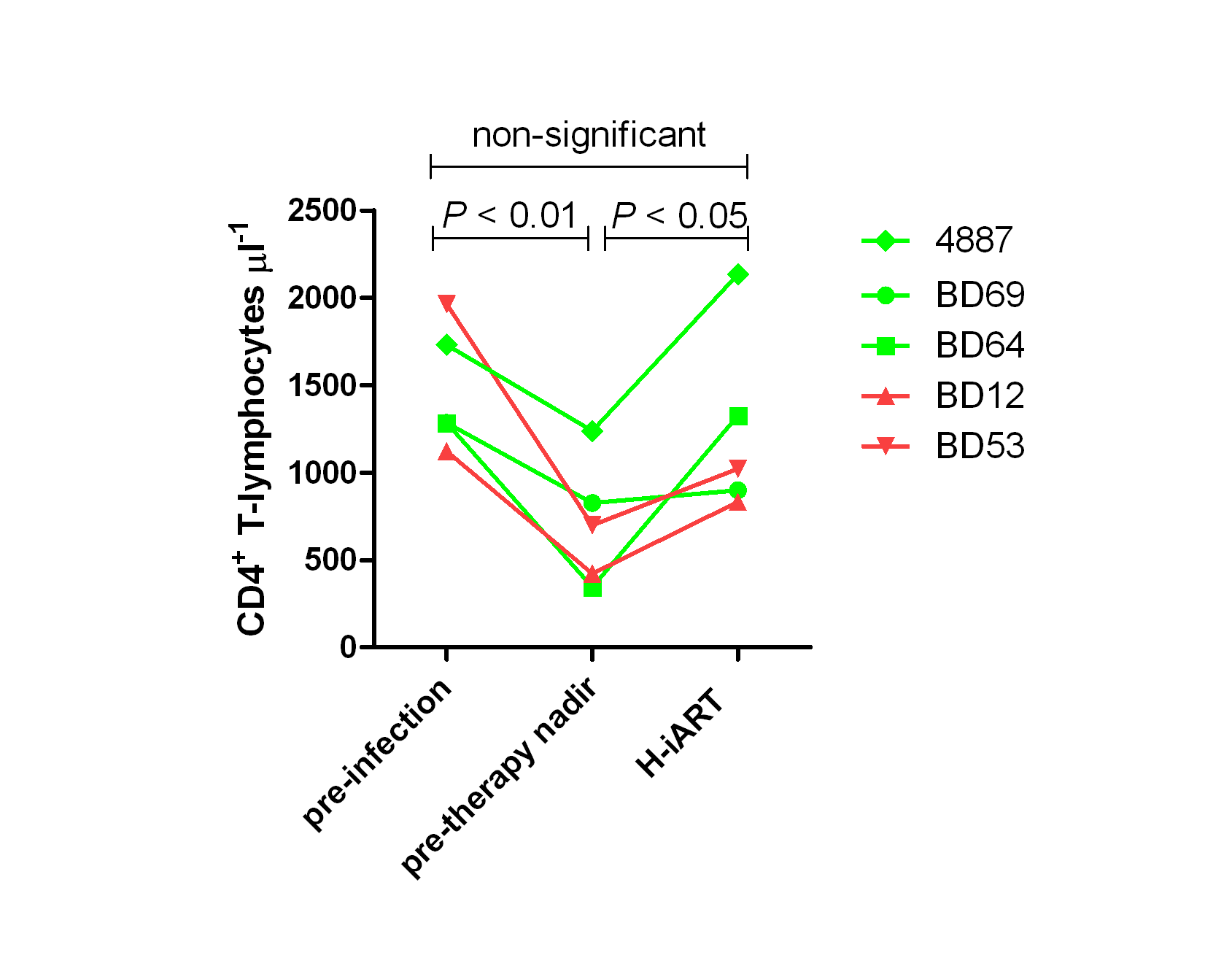

Supplement: Figure S6 — Treatment with H-iART recovers CD4+ T-cell counts decreased by pathogenic SIVmac251 infection. Five macaques are considered for which pre-infection and pre-treatment CD4+ T-cell counts were available. Values during H-iART refer to a median period of 89 days (range: 83–89 days). Data have been analyzed using one-way ANOVA followed by Newmann-Keuls test. (TIF) [file ppat.1002774.s006.tif]

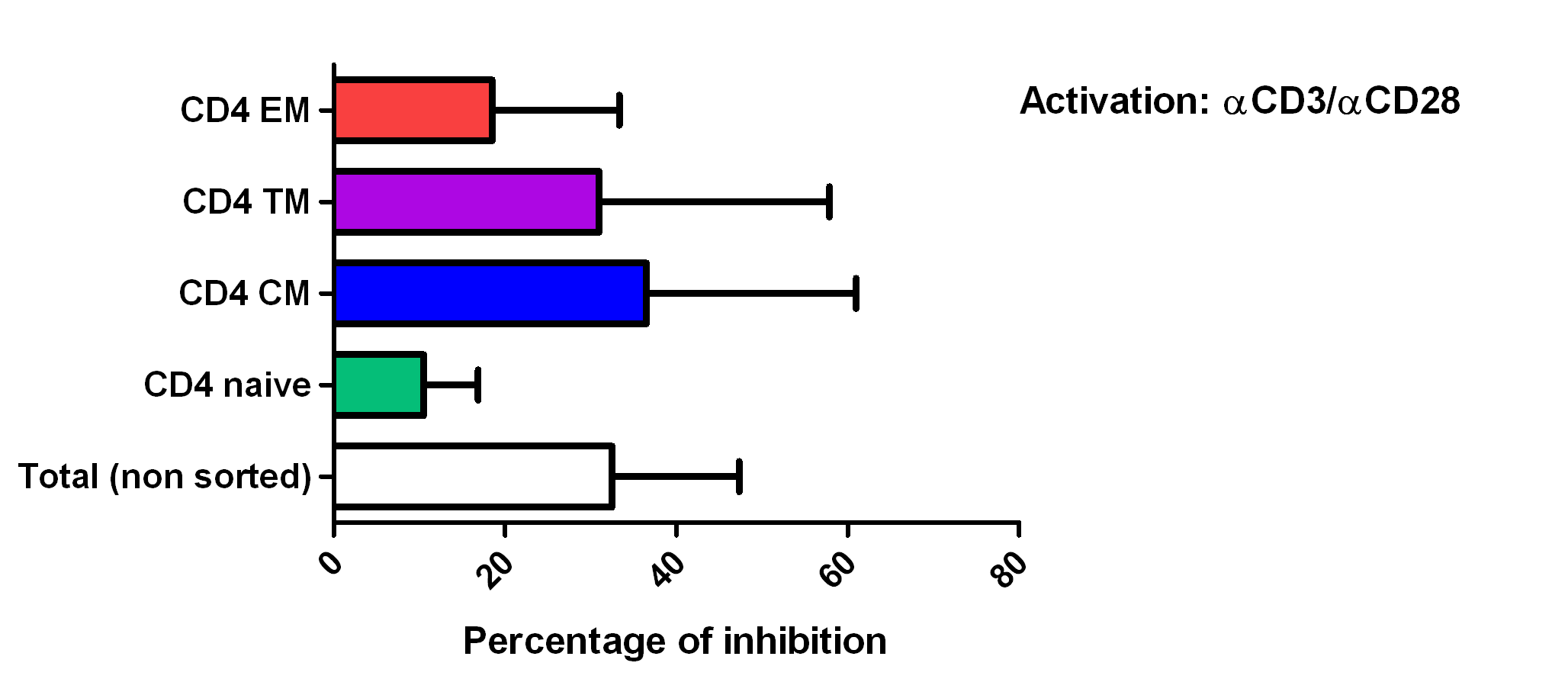

Supplement: Figure S7 — Maraviroc decreases T-cell proliferation in vitro . The percentage inhibition of proliferation induced by 0.1 µM MRV in CD4+ T-cells activated with αCD3/αCD28 is shown. Data are shown as mean + SEM and are derived from two experiments. CM: central memory; TM: transitional memory; EM: effector memory. P = 0.0417; Friedman's test. (TIF) [file ppat.1002774.s007.tif]

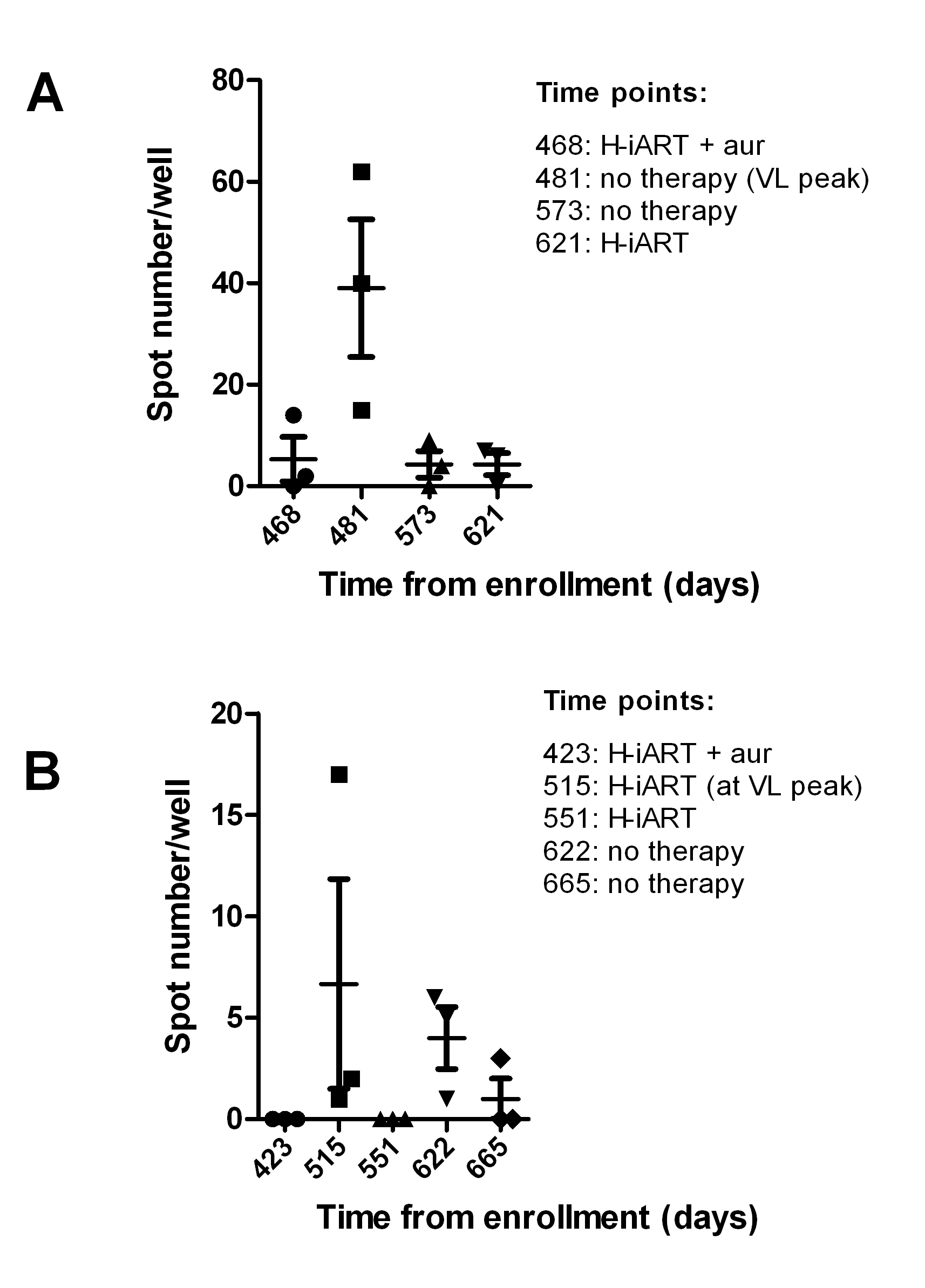

Supplement: Figure S8 — ELISPOT analysis of the number of interferon-γ secreting cells/1.5 * 105 PBMCs. The analyses refers to A: macaque P252 and B: macaque P177. The time points selected are shown as days from the zero point adopted in figure 8 of the main text. (TIF) [file ppat.1002774.s008.tif]

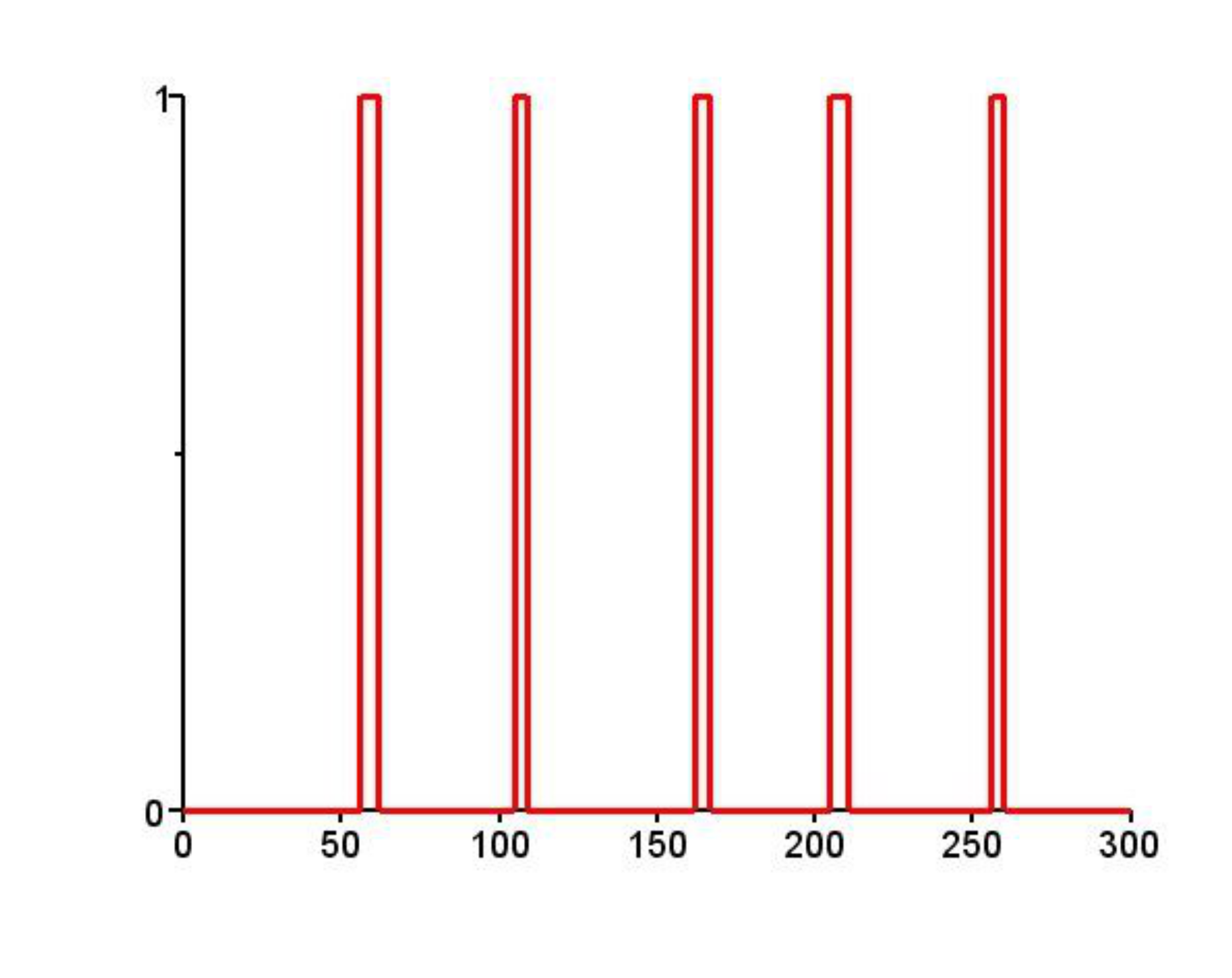

Supplement: Figure S9 — Activation function. The step function f(t) which determines the activation of resting latently infected CD4+ T-cells. Times between two activation periods follow a Poisson distribution with a mean of 50 days. The length of activation periods follows a uniform distribution over an interval of 4 to 6 days. (TIF) [file ppat.1002774.s009.tif]

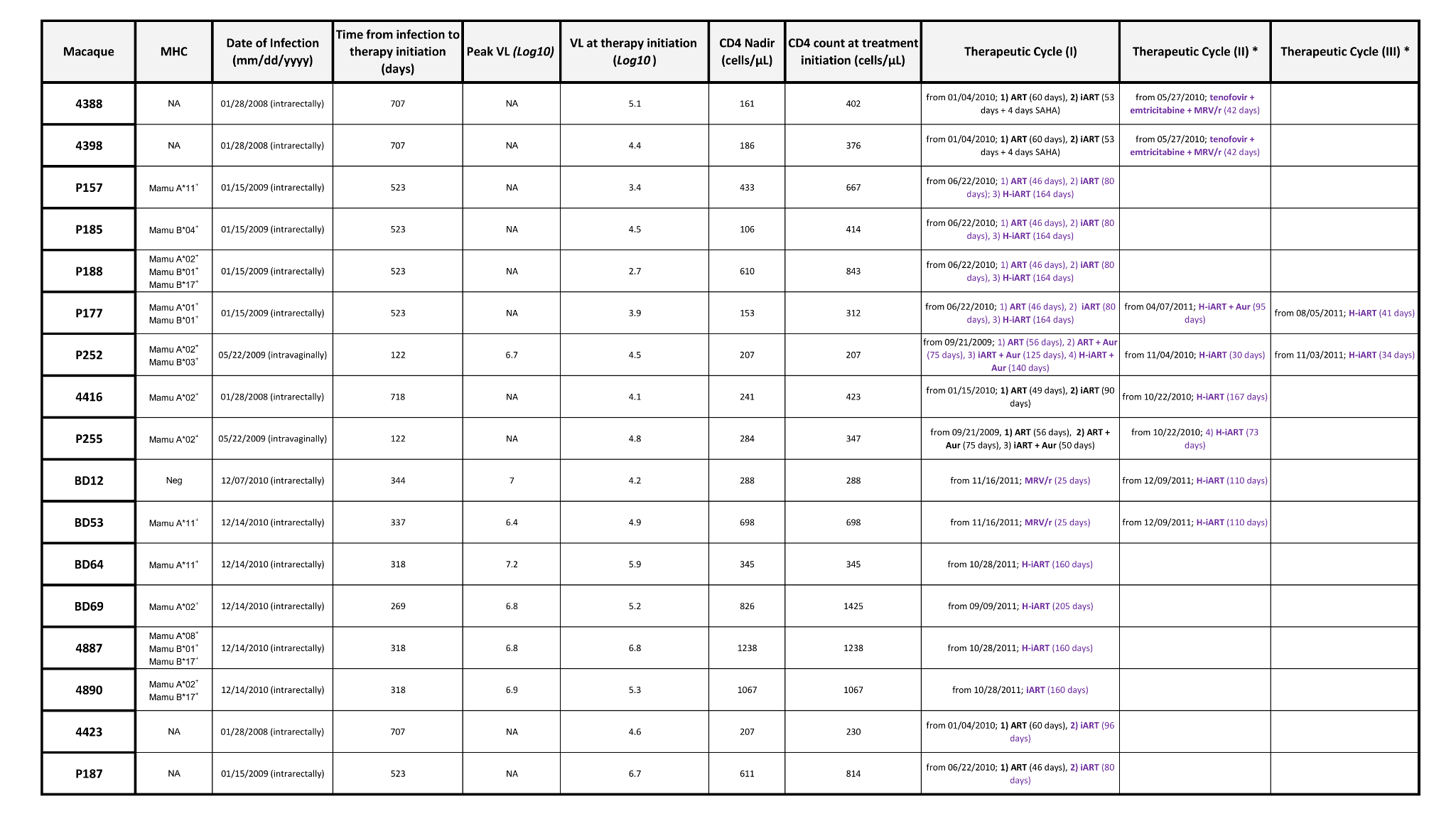

Supplement: Table S1 — Viro-immunological background and therapeutic regimens of the SIVmac251 infected macaques employed in the study. MHC alleles analyzed are the following: Mamu A*01; A*02; A*08; A*11; B*01; B*03; B*04; B*08; B*17. The CD4 nadir is chosen as the lowest pre-therapy T-CD4 count available. Therapeutic regimens described in the present article are highlighted in violet. (TIF) [file ppat.1002774.s010.tif]

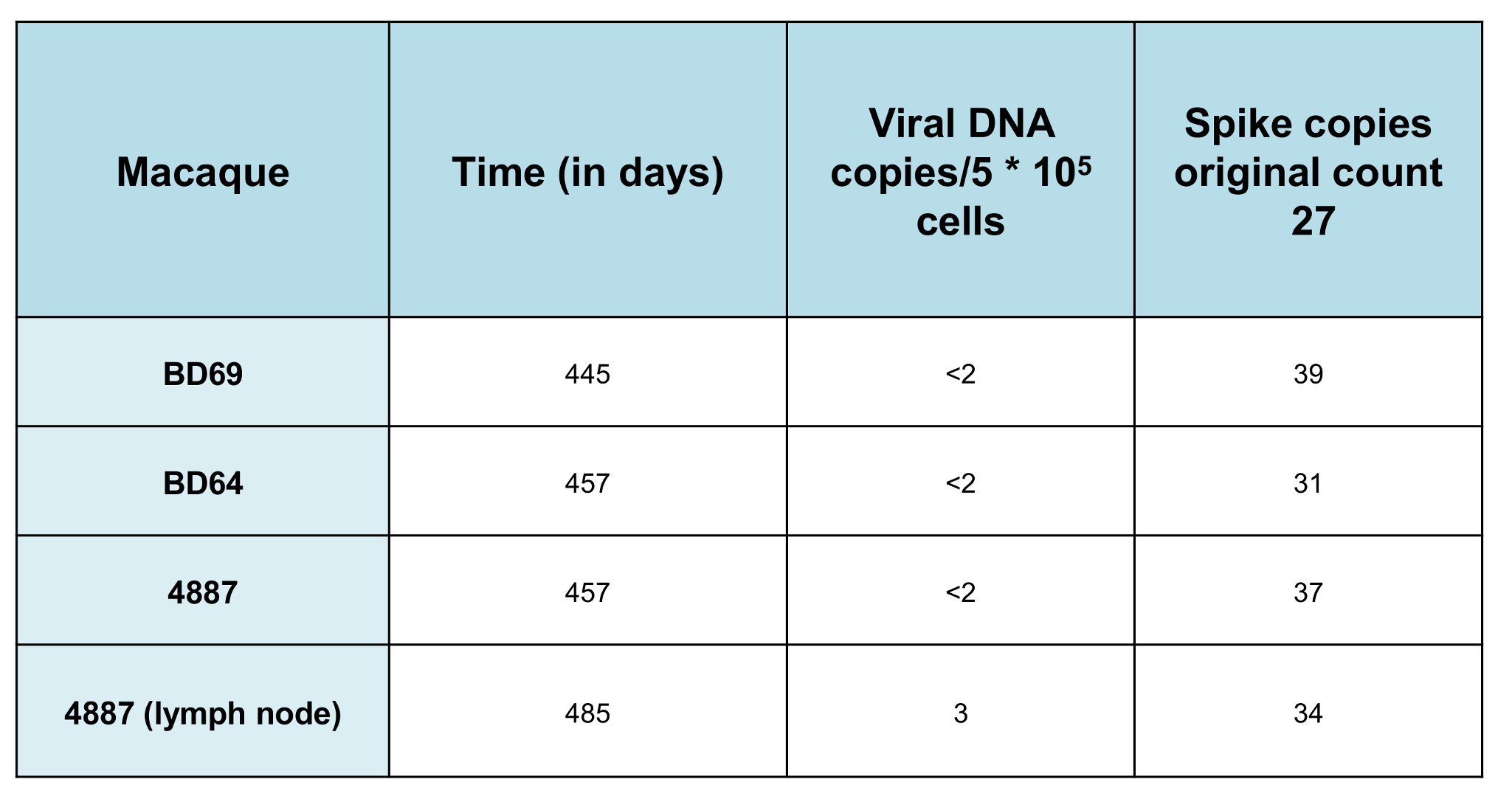

Supplement: Table S2 — Validation of the real-time PCR assay for SIVmac251 DNA quantification in PBMCs and lymph node biopsies. The limit of detection of the assay is 2 copies/5*105 cells. As a control of the assay variability and to exclude PCR inhibition, spiked DNA measurements for each sample were used. (TIF) [file ppat.1002774.s011.tif]

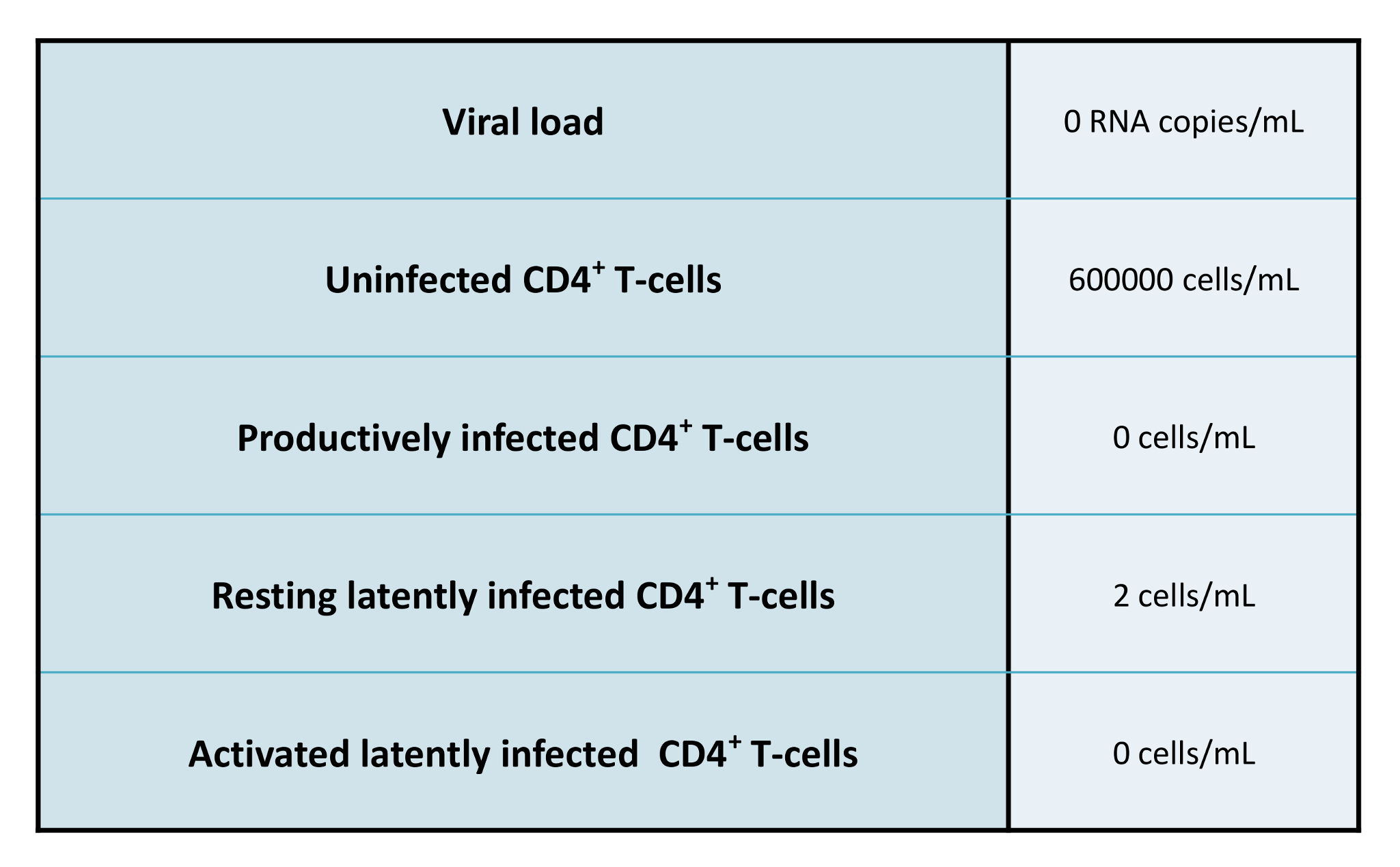

Supplement: Table S3 — Starting data for the numerical simulations of the viral load and reservoir dynamics. (TIF) [file ppat.1002774.s012.tif]
