# Supplementary material for: A Highly Intensified ART Regimen Induces Long-Term Viral Suppression and Restriction of the Viral Reservoir in a Simian AIDS Model
Source: PLoS Pathog. 2012 Jun 21;8(6):e1002774. doi: 10.1371/journal.ppat.1002774 (PMC3380955; doi:10.1371/journal.ppat.1002774)
Supplement: Text S1 — Mathematical modeling and numerical simulations. (DOCX) [file ppat.1002774.s013.docx]

**Text S1. Mathematical modeling and numerical simulations**

Numerical simulations used in the paper are based on the nonlinear system of ordinary differential equations (4) in [1]:


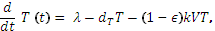

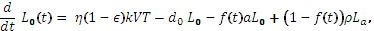

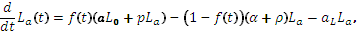

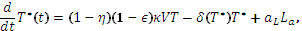

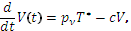


where represents CD4^+^ T cells susceptible to infection, represents productively infected cells, and represent respectively resting and activated latently infected cells and represents the viral load. For the exhaustive definitions and values of all parameters see Table 1 in [1].


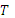

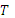

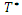

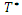

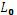

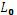

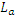

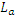

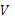

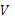


The main purpose of the model is to provide a quantitative interpretation which correlates activation of cells of the latent reservoir and observable viral blips. The model simulates viral load peaks of reasonable amplitude and duration by means of the introduction of a random activation step function → , where and are the times at which the simulation begins and ends. *f* is used as a coefficient for the terms concerning the activation of latently infected cells: when *f* takes value 0 there is no activation, while when *f* takes value 1 then a fraction *a* of latently infected cells is activated in unit of time (1 day). The complement is used as a coefficient for the terms which account for reversion to resting state of activated latently infected cells, so that when *f* takes value 0 (*i.e.* when there is no activation) a fraction *ρ* of activated latently infected cells returns to resting state in the unit of time.


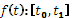

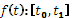

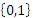

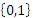

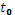

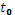

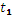

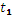

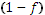

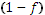


According to [1], times between two periods of activation follow a Poisson distribution with a mean value of 50 days, while the length of the activation periods obeys a uniform distribution over the interval of 4 to 6 days, which leads to viral blips that are in good agreement with experimental observations [1]. The activation function used in the simulations, generated with the RANDLIB package of the Scilab 5.3.3 software, is shown in figure S8.

Simulations relative to the macaque model required a higher burst size () than in the human model (4) in [1], which according to [2] was assumed to be 55000 RNA copies per CD4^+^ T-cell. Moreover, a higher death rate for productively infected CD4^+^ T cells () was also required, and according to our experimental data (not shown) it was assumed to be 7.20 . The values of all other parameters were chosen according to [1].


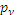

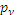

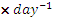

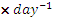

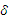

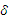

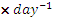

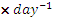


In the model, half-life of latent reservoir is significantly dependent on the proliferation rate of activated CD4^+^ T-cells. With *p* = 1.4 no decay is observable in latent reservoir with either 0.95 or 0.99 drug efficacy () rates, while with *p* = 0.945 a slow decay is observable (see figure 9D). Half-life of latent reservoir is also dependent from the mean value of the Poisson distribution of times between two activation periods in the activation step function *f*. With our mean value (50 days), numerically estimated half-life over a sample of 50 simulations with starting values of ranging from 1 to 20 cells/mL (data not shown) is about 200 days. This suggests that, with given values for proliferation and drug efficacy rates, an average number of 4 periods of activation of resting latently infected CD4^+^ T cells is needed to halve the size of the latent reservoir. For a description of the numerical analysis methods see the “Materials and Methods” section (Statistical and biomathematical analyses).


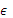

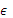

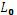

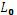


**References:**

1. Rong L, Perelson AS. (2009) Modeling latently infected cell activation: viral and latent reservoir persistence, and viral blips in HIV-infected patients on potent therapy. PLoS Comput Biol. 5(10):e1000533
2. Chen HY, Di Mascio M, Perelson AS, Ho DD, Zhang L. (2007) Determination of virus burst size in vivo using a single-cycle SIV in rhesus macaques. Proc Natl Acad Sci U S A.;104(48):19079-84.
