# Supplementary material for: A Highly Intensified ART Regimen Induces Long-Term Viral Suppression and Restriction of the Viral Reservoir in a Simian AIDS Model
Source: PLoS Pathog. 2012 Jun 21;8(6):e1002774. doi: 10.1371/journal.ppat.1002774 (PMC3380955; doi:10.1371/journal.ppat.1002774)
Supplement: Text S2 — Bioinformatic analyses and molecular modeling studies. (DOCX) [file ppat.1002774.s014.docx]

**Text S2. Bioinformatic analyses and molecular modeling studies**

Sequence alignments were conducted using the VAST option in the NCBI database, in case three-dimensional structures were available, or using CLUSTALW [<http://www.ebi.ac.uk/tools/msa/clustalw2>]. Amino acids susceptible to drug resistance mutations were retrieved from the Stanford Drug Resistance Database [<http://hivdb.stanford.edu/>].

For structural alignments aimed at molecular modeling, structures were retrieved from the Protein Data Bank, and structural alignments were performed using the Swiss PDB Viewer (SPDBV) program (Swiss Institute of Bioinformatics). Briefly, the α-carbons of the highly conserved DTG motifs (*i.e.* D25, T26 and G27 for HIV-1 protease) were initially superimposed using the “fit molecules” option. Then using the “improve fit” option, SPDBV was asked to minimize the root-mean square deviation (RMSD) between the corresponding atoms using a least square algorithm. Using the default matrix embedded in the program (with open and extended gap penalties of 6 and 4, respectively), the calculation was extended to neighboring atoms until the maximum number of aligned atoms with the lowest RMSD was obtained. Then, the “colour-alignment diversity” option was used. Homology models were generated using the “SWISS Model” website [<http://swissmodel.expasy.org/>], following alignments of the protein sequences using CLUSTALW. SPDBV was employed to color the 3D structures by alignment diversity.

To investigate the energetics of DRV bound to HIV-1 and HIV-2 proteases, DRV/HIV-1 protease and DRV/HIV-2 protease complexes were studied by molecular dynamics simulation. The crystal structure of SIVmac251 protease in complex with inhibitor SB203386 (PDB 1TCW [1]) was superimposed to the crystal structure of HIV-2 protease in complex with darunavir (DRV) (PDB 3EBZ [2]) using the “protein structure alignment” tool in Maestro (Maestro, version 9.2, Schrödinger, LLC, New York, NY, 2011.). Water molecules, ions and cofactors were removed from the two structures, and SB203386 in 1TCW was replaced by DRV in its 3EBZ-bound conformation. The complexes were then relaxed using the Macromodel program (MacroModel, version 9.9, Schrödinger, LLC, New York, NY, 2011) through 10 ps of molecular dynamics (MD) simulations, followed by 100 steps of Polak-Ribiere conjugate gradient minimization, both using MM-GB/SA as solvation treatment [3]. The resulting structures were submitted to MD calculations in explicit solvent, using the TIP4P water model [4] in a Periodic Boundary Conditions cubic box. Desmond molecular Dynamics System version 2.4 (D. E. Shaw Research, New York, NY, 2010; [5]), was used to setup and run the MD simulations. The systems to be simulated were built using the “system builder” utility with the structures being neutralized by Na^+^ and Cl^-^ ions. The buried regions were solvated using the “solvate pocket” utility. Before performing the simulation, a series of minimizations and short MD simulations were carried out to relax the model system, by means of a relaxation protocol which consists of six stages:

1. Minimization with the solute restrained;

2. Minimization without restraints;

3. Simulation (12 ps) in the NVT ensemble using a Berendsen thermostat (10 K) with non-hydrogen solute atoms restrained;

4. Simulation (12 ps) in the NPT ensemble using a Berendsen thermostat (10 K) and a Berendsen barostat (1 atm) with non-hydrogen solute atoms restrained;

5. Simulation (24 ps) in the NPT ensemble using a Berendsen thermostat (300 K) and a Berendsen barostat (1 atm) with non-hydrogen solute atoms restrained;

6. Unrestrained simulation (24 ps) in the NPT ensemble using a Berendsen thermostat (300 K) and a Berendsen barostat (1 atm);

At this point, 20-ns long simulations were carried out in the NPT ensemble using a Berendsen thermostat (300 K) and a Berendsen barostat (1 atm). All of the molecular mechanics calculations have been carried out using an OPLS force-field.[6,7] MD results were analyzed using the simulation event analyzer embedded in Desmond/Maestro. Interaction energies and bound conformations of DRV within the binding pocket, averaged over the 20-ns simulations, resulted almost the same for both complexes (ΔE =~ 1.5 kcal/mol);the number of direct hydrogen bonds between DRV and each of the two enzymes during the simulation is comparable as well (SIV = 3.6, HIV-2 = 3.4).

**References**:

1) Hoog SS, Towler EM, Zhao B, Doyle ML, Debouck C, Abdel-Meguid SS (1996) Human immunodeficiency virus protease ligand specificity conferred by residues outside of the active site cavity. Biochemistry 35:10279-10286

2) Kovalevsky AY, Louis JM, Aniana A, Ghosh AK, Weber IT (2008) Structural evidence for effectiveness of darunavir and two related antiviral inhibitors against HIV-2 protease. J.Mol.Biol. 384: 178-192.

3) Still WC; Tempczyk A; Hawley RC; Hendrickson T (1990) Semianalytical treatment of solvation for molecular mechanics and dynamics J. Am. Chem. Soc. 112 6127-6129.

4) Jorgensen WL, Chandrasekhar J, Madura JD, Impey RW, Klein ML, J. (1983) Chem. Phys. 79, p. 926.

5) Bowers KJ, Chow E, Xu H, Dror RO, Eastwood MP et al. (2006) Scalable Algorithms for Molecular Dynamics Simulations on Commodity Clusters. Proceedings of the ACM/IEEE Conference on Supercomputing (SC06), Tampa, Florida,

6) Jorgensen WL, Maxwell DS, Tirado-Rives J (1996) Development and Testing of the OPLS All-Atom Force Field on Conformational Energetics and Properties of Organic Liquids. J. Am. Chem. Soc. 118 (45): 11225–11236.

7) Jorgensen WL, Tirado-Rives J (1988). The OPLS Force Field for Proteins. Energy Minimizations for Crystals of Cyclic Peptides and Crambin. J. Am. Chem. Soc. 110: 1657–1666.
