# Supplementary material for: A Highly Intensified ART Regimen Induces Long-Term Viral Suppression and Restriction of the Viral Reservoir in a Simian AIDS Model
Source: PLoS Pathog. 2012 Jun 21;8(6):e1002774. doi: 10.1371/journal.ppat.1002774 (PMC3380955; doi:10.1371/journal.ppat.1002774)
Supplement: Text S3 — Retrospective analysis of the response of SIV-mac251 to maraviroc in vivo . (DOCX) [file ppat.1002774.s015.docx]

**Text S3. Retrospective analysis of the response of SIV-mac251 to maraviroc *in vivo***.

Two monkeys (4388 and 4398), were put under a regimen of tenofovir (20 mg/kg/day), emtricitabine (50 mg/kg/day), and maraviroc (MRV; 50 mg bid; chosen on the basis of body surface calculations) boosted by ritonavir (50 mg bid). This therapy was administered 10 weeks after the suspension of a previous regimen (see ref. 1) that failed to control viremia in both animals.

Results showed that viral loads fell to levels significantly lower than those displayed before the treatment (Fig. S3). On week 3 of treatment, again, viral load showed an increasing trend in one animal; therefore, the MRV dosage was doubled in an ultimate attempt to regain control of viral load. While one monkey maintained a detectable viral load, the other monkey responded with an undetectable viral load at 5 weeks (Fig. S3).

Although monkeys were not treated with MRV alone, there is evidence supporting the contribution of MRV to the antiretroviral effects observed:

1) Retrospective genotypic analysis of the virus in frozen plasma samples showed the presence of resistance mutations for both tenofovir (K65R, detected in 7/19 clones in macaque 4388 and in 20/20 clones in macaque 4398) and emtricitabine (M184V, detected in 9/19 clones in macaque 4388).

2) Neither of the monkeys had shown an undetectable viral load after the emergence of drug resistance mutations during the previous therapeutic cycle, despite maintenance of an antiretroviral regimen containing both drugs that were then associated with MRV.

**References:**

1. Lewis MG, Dafonseca S, Chomont N, Palamara AT, Tardugno M et al. (2011) Gold drug auranofin restricts the viral reservoir in the monkey AIDS model and induces containment of viral load following ART suspension. AIDS. 25(11):1347-56
