# Supplementary material for: A Highly Intensified ART Regimen Induces Long-Term Viral Suppression and Restriction of the Viral Reservoir in a Simian AIDS Model
Source: PLoS Pathog. 2012 Jun 21;8(6):e1002774. doi: 10.1371/journal.ppat.1002774 (PMC3380955; doi:10.1371/journal.ppat.1002774)
Supplement: Text S4 — In vitro measurement of the effect of MRV on T-cell proliferation. (DOCX) [file ppat.1002774.s016.docx]

**Text S4. *In vitro* measurement of the effect of MRV on T-cell proliferation**

For measuring the in vitro effects of MRV on cell proliferation, CD4^+^ T-cells were isolated by positive selection (MACS Separation Columns, Miltenyibiotec) (purity: >90%). Purified CD4^+^ T-cells were incubated (1.5 x 10^5^cells /well) with α-CD3 and α-CD28 antibodies in 96-well coated plates previously prepared overnight at 4°C, treated and mock-treated with increasing concentrations of MRV, ranging from 0.1-1 µM for 24 hours in an incubator at 37°C with 5% CO_2._ After 24 h, cells were harvested and resuspended in PBS 1x with 5% FBS and stained in cytofix and cytoperm solution (BD) containing 1:100 of propidium iodide for 30’ at 4°C. The cells were then washed 2 times with 1x BD Perm/Wash solution and resuspended in 200 µl PBS1x with 5% FBS prior acquisition. 200.000 events were collected during flow cytometric analysis on a FACSCalibur flow cytometer and analyzed using the CellQuest Pro software(BD). For analyses on CD4^+^ T-cell subsets, isolated CD4^+^ T-cells were stained for 20 minutes at 4°C in Macs buffer PBS-1,25% FBS, 2mM EDTA) with the following combination of antibodies: α-CD45RO-FITC-conjugated antibody (Becton Dickinson (BD), Franklin Lakes, NJ), α-CD27-PE-conjugated antibody (Miltenyi Biotec, Bergisch Gladbach, Germany), α-CCR7-PE Cy7-conjugated antibody (BD). Cells were washed in MACS buffer before being resuspended at 30 x 10^6^ cells/mL in the same buffer for sorting. Naive CD4^+^ T-cells (TN), TCM, TTM and TEM cells (purity: >98%) were sorted on an FACS Aria cell sorter (Becton Dickinson) at low pressure equipped with an automatic cloning deposition unit. Cells were selected on the basis of physical parameters and fluorescence and collected in on sterile tubes. Sorted cells were analyzed for purity by flow-cytometry with a FACSCanto flow-cytometer (BD). The sorted cells were resuspended at a concentration of 2 x 10^5^/mL and incubated with α-CD3, α-CD28 coated plates. The human cell subpopulations were also incubated with 0.1 μM of MRV. After 24h, cells were harvested and washed in PBS 1x with 5% FBS and stained in cytofix and cytoperm solution (BD) containing 1:100 of propidium iodide for 30’ at 4°C. The cells were then washed 2 times with 1x BD Perm/Wash solution and resuspended in 200 µl PBS 1x with 5% FBS prior acquisition. 200.000 events were collected during flow cytometric analysis on a FACSCalibur flow cytometer and analyzed using the CellQuest Pro software(BD).
